# Harnessing Natural Sequence Variation to Dissect Posttranscriptional Regulatory Networks in Yeast

**DOI:** 10.1534/g3.114.012039

**Published:** 2014-06-17

**Authors:** Mina Fazlollahi, Eunjee Lee, Ivor Muroff, Xiang-Jun Lu, Pilar Gomez-Alcala, Helen C. Causton, Harmen J. Bussemaker

**Affiliations:** * Department of Biological Sciences, Columbia University, New York, New York 10027; § Electrical Engineering Department, Columbia University, New York, New York 10027; † Department of Systems Biology, Columbia University, New York, New York 10032; ‡ present address: Genetics and Genomic Sciences, Mount Sinai Hospital, New York, New York 10029

**Keywords:** RNA-binding proteins, *cis*-regulatory analysis, inference of protein-level regulatory activity, quantitative trait locus (QTL) mapping, Pumilio/FBF homology domain proteins (Puf3p Puf4p)

## Abstract

Understanding how genomic variation influences phenotypic variation through the molecular networks of the cell is one of the central challenges of biology. Transcriptional regulation has received much attention, but equally important is the posttranscriptional regulation of mRNA stability. Here we applied a systems genetics approach to dissect posttranscriptional regulatory networks in the budding yeast *Saccharomyces cerevisiae*. Quantitative sequence-to-affinity models were built from high-throughput *in vivo* RNA binding protein (RBP) binding data for 15 yeast RBPs. Integration of these models with genome-wide mRNA expression data allowed us to estimate protein-level RBP regulatory activity for individual segregants from a genetic cross between two yeast strains. Treating these activities as a quantitative trait, we mapped *trans*-acting loci (activity quantitative trait loci, or aQTLs) that act via posttranscriptional regulation of transcript stability. We predicted and experimentally confirmed that a coding polymorphism at the *IRA2* locus modulates Puf4p activity. Our results also indicate that Puf3p activity is modulated by distinct loci, depending on whether it acts via the 5′ or the 3′ untranslated region of its target mRNAs. Together, our results validate a general strategy for dissecting the connectivity between posttranscriptional regulators and their upstream signaling pathways.

The advent of high-throughput genotyping and gene expression profiling technologies has made it possible to treat messenger RNA levels as quantitative traits and map the *cis*- and *trans*-acting loci that drive their variation (Brem *et al.* 2002; Schadt *et al.* 2003; Smith and Kruglyak 2008; Lee *et al.* 2009). This has created new opportunities for studying genetic variation at the level of gene regulatory networks rather than individual genes. A common approach is to treat the mRNA expression levels as heritable traits and use them to identify expression quantitative trait loci (eQTL) hotspots that regulate the expression of a large number of genes. Detection of eQTL hotspots is then based on a clustering procedure that identifies loci with many significant eQTL linkages.

Integrative studies thus far have focused on regulation by DNA-binding transcription factors (Ye *et al.* 2009; Lee and Bussemaker 2010). However, posttranscriptional control of transcript stability by RNA-binding proteins (RBPs) is also critical for the regulation of mRNA abundance (Foat *et al.* 2005). An increasing number of studies report the involvement of posttranscriptional regulation by RBPs in human genetic disorders (Lukong *et al.* 2008; Cooper *et al.* 2009; Polymenidou *et al.* 2012; Yamazaki *et al.* 2012), and several studies have identified nucleotide motifs associated with posttranscriptional regulation by RBPs (Foat *et al.* 2005; Shalgi *et al.* 2005; Hogan *et al.* 2008; Riordan *et al.* 2011).

Even though many studies have shown that mRNA stability is often regulated through *cis*-regulatory elements in the 3′ untranslated region (UTR), examples also exist of stability regulation through the 5′ UTR of the transcripts, such as an upstream open reading frame (ORF) that can inhibit ribosomal scanning and promote mRNA decay (Vilela *et al.* 1999; Hatano *et al.* 2013). It has also been shown that binding by the heat shock protein HSP70 to a motif located in the 5′ UTR of the tumor suppressor gene *SMAR1* stabilizes the transcript and leads to increased SMAR1 protein levels (Pavithra *et al.* 2010). Finally, the secondary structure of the 5′ UTR has been linked to the mRNA stability (Cannons and Cannon 2002). Taken together, these studies show that it is important to include the 5′ UTR when searching for *cis*-regulatory elements that control mRNA stability and not just focus on the 3′ UTR.

In this article, we formulate and apply a general method for discovering genetic polymorphisms that accounts for differences in genome-wide mRNA abundance patterns between strains or individuals that reflect posttranscriptional regulation of mRNA stability. Because our approach uses the RNA sequence specificity of RBPs as prior information, we first systematically constructed sequence-to-affinity models for 15 RBPs. Our motif discovery procedure uses the MatrixREDUCE algorithm, which combines accurate biophysical modeling of protein−RNA interaction (Foat *et al.* 2005, 2006) with the use of high-throughput *in vivo* mRNA binding data (Riordan *et al.* 2011). As previously demonstrated, the resulting position-specific affinity matrices (PSAMs) can be used to infer changes in the protein-level regulatory activity of each RBP from the genome-wide pattern of changes in steady-state mRNA level (Foat *et al.* 2005).

For the systems genetics component of our study, we exploited parallel mRNA expression data and genotype data across a segregating population of yeast strains. The goal was to identify chromosomal loci that modulate the protein-level regulatory activity of a particular RBP. To this end, we first inferred RBP activities for each segregant from its genome-wide mRNA expression pattern. Next, we treated the activity of each particular RBP as a quantitative trait, and used linkage analysis to map genetic loci whose allelic variation has an effect on the RBP activity. We call these loci activity quantitative trait loci, or “aQTLs,” by analogy with a study on DNA-binding transcription factors (Lee and Bussemaker 2010). Because we infer the RBP activities from the collective behavior of the targets of each particular RBP, our trait is far less noisy than the expression levels used as traits in eQTL analyses. Also because of the reduced number of marker/trait combinations that needs to be tested for significance compared with eQTL analyses, the statistical power to detect *trans*-acting genetic variation is greatly increased.

Our analysis yielded causal aQTL relationships for a number of RBPs, including Puf3p and Puf4p, members of the Pumilio/FBF (PUF) homology domain family. The regulatory activities of these factors typically change in opposite directions in response to changes in nutrient conditions, suggesting regulation by a single genetic locus. However, we found that the activity of these two PUF proteins are linked to distinct genetic loci. We mapped distinct aQTLs depending on whether Puf3p acts via binding to the 5′ or to the 3′ UTR of its target mRNAs. We also performed experiments to determine the effect of allelic variation at the *IRA2* locus on Puf4p posttranscriptional activity. We found that the activity of Puf4p depends on the allele at the *IRA2* locus in the BY but not the RM strain background. It is likely that other polymorphisms in RM make expression of Puf4p targets less sensitive to Puf4p activity.

## Materials and Methods

### Strain construction

The *PUF4*::*KanMX* cassette was amplified from the relevant *Mata* strain from the *Saccharomyces cerevisiae* Genome deletion collection (sequence-www.stanford.edu/group/yeast_deletion_project/deletions3.html) and used to transform BY4716 (*MATα lys2Δ0)* and YLK807 (*MATa lys2Δ0 ura3Δ0 IRA2RM)*. G418 (Geneticin; Life Technologies, Grand Island, NY)-resistant cells were selected and the site of integration confirmed by polymerase chain reaction (PCR). For RM1-11a (*MATa leu2Δ0 ura3Δ0 ho*::*KanMX)* and YLK810 *Mata leu2Δ0 ura3Δ0 ho*::*KanMX IRA2BY)*, the KanMX at the *ho* locus was replaced with the NatMX cassette by transformation with p4339 cut with *Eco*RI (Tong *et al.* 2001). Nourseothricin-resistant, Geneticin-sensitive transformants were selected and transformed with the *PUF4*::*KanMX* amplicon, as described previously (see Supporting Information, Table S4 for details).

### Real-time (RT)-PCR

Yeast were grown under standard laboratory conditions to early log phase in yeast extract peptone dextrose (YPD) and harvested by centrifugation. Total RNA was extracted using the Master Pure YeastRNA Purification Kit (Epicentre, Madison, WA) and 2.5 ng was used to generate cDNA with the iScript cDNA Synthesis Kit (Bio-Rad, Hercules, CA). 1/20^th^ of this reaction mix was used as template for RT-PCR using 1 × iQSYBR Green Supermix (Bio-Rad, Hercules, CA). All reactions were carried out in triplicate using a StepOnePlus machine (Applied Biosystems, Foster City, CA) and the data analyzed using AB software. Three technical replicates were carried out for each strain (see Figure S6).

Amplification efficiencies were determined for each set of primers using serial dilutions of genomic template from 8 to 0.25 ng/µL. Relative amounts of *RRS1* were determined, using *THI6* as a reference (Pfaffl 2001). Each cDNA synthesis reaction was carried out in parallel with a control sample lacking reverse transcriptase, so that the signal due to contaminating genomic DNA could be determined.

### Primers used for RT-PCR

For *RRS1*, we used the primers 5′-GGT AAC CTG GCA GCA TTC GA-3′ and 5′-TGC AAC AAG GTC ATC ACG GA-3′. For*THI6*, we used 5′-GGT GTA GAA CCG TTG GTA TTG G-3′ and 5′-CTG GTA GCA TCT AAC AAG CCT CTC-3′.

### RBP data set

For our motif search, we analyzed genome-wide imunoaffinity purification data for 45 different RNA binding proteins (Hogan *et al.* 2008). In this study, bound mRNA molecules were isolated at mid-log phase from cells growing in YPD media. The mRNA was hybridized to a microarray. For each RBP, two to six experimental replicates were performed for a total of 132 immunoprecipitation (IP) experiments.

### Segregant mRNA expression and genotype data sets

For the aQTL analysis, we used genome-wide mRNA expression data for 108 haploid segregants from a genetic cross between two parental strains: BY4716 and RM11-1a (Smith and Kruglyak 2008). As differential expression values, we used the log_2_-ratios between segregants and a reference consisting of a mixture of the BY and RM strains. Genotype data for the same segregants at 2956 markers was obtained (Brem *et al.* 2002).

### Preprocessing of RBP binding data

For our motif analysis, we took log_2_-ratios between the microarray intensities for the immunoprecipitated sample and input sample, respectively, for each RBP. To reduce the effect of outliers, we applied a rank-quantile transformation based on the standard normal distribution. For each RBP, $x=(x_{1},x_{2},\ldots ,x_{n})$ denotes the vector of binding log_2_-ratios across all genes, sorted in ascending order. We first ranked the data points (*x*) in each column (i.e. IP experiment). Let Pr(X<χ) denote the cumulative distribution function for a standard normal random variable *X*. As illustrated in Figure S1, we then defined $\chi_{i}$ as the i^th^ quantile,

$$\text{Pr}(X<\chi_{i})=\frac{\text{rank}(x_{i}-\frac{1}{2})}{n}$$

and used it to replace the i^th^ element in the vector *x*. This transformation reduces the effect of outliers.

### Motif discovery

To model for RBP-mRNA binding occupancy, we used a biophysical model similar to that presented by Foat *et al.* (2006). We assume that the free protein concentration is low relative to the dissociation constant for protein-RNA interaction (Ghaemmaghami *et al.* 2003; Miller *et al.* 2008; Zhu *et al.* 2009). The occupancy (*N*) of sequence (*S*) of mRNA of gene (*g*) by an RBP (*ϕ*) is given as follows:

$$N_{g\phi }(S)\approx [\phi ]K_{g\phi }(S)$$

Where the total affinity $K_{g\phi }$ of *S* is defined as the sum of the relative affinities of a sliding window of length $L_{\phi }$. Here, $L_{\phi }$ represents the length of the binding site.

$$K_{g\phi }=\sum_{i\in S}\prod_{j=1}^{L_{\phi }}W_{\phi jb_{i+j-1}}(S)$$

Here *b* indicates the nucleotide identity of the base at the coordinate i+j−1 within sequence *S*. The aforementioned formula assumes that the contribution to the binding free energy at each position within the binding site is independent. The set of w’s thus represent the PSAM of RBP (*ϕ*).

We assume the relative abundances between the amount of mRNA bound to an RBP and the control sample is approximately proportional to the mRNA occupancy. We can then use the binding data to train the PSAMs (Foat *et al.* 2005). Our RBPs motif discovery approach is shown in Figure S2. To detect the motifs, we used the MatrixREDUCE program from the REDUCE Suite package (bussemakerlab.org/software/REDUCE) to perform a genome-wide fit of a PSAM to the rank-quantile log_2_-ratios of RBP binding data. MatrixREDUCE builds a multiple linear regression model originally developed by (Foat *et al.* 2005, 2006). We used an enhanced version of MatrixREDUCE that can infer PSAMs and can explain the signal variation in multiple data sets simultaneously.

The MatrixREDUCE algorithm consists of two steps: seed motif finding and PSAM optimization. The seed finding step seeks to identify within the set of all possible nucleotides of a specified length, the one whose occurrence best correlates with the binding signal. The motif size is allowed to vary from to 1 to 10 nucleotides in our analysis. Once the optimal motif was identified, it was used as a seed for the optimization procedure. For an optimal motif of length *L*, a matrix of size 4×L, representing each nucleotide A, C, G, and T/U at positions 1 to *L* was constructed. At every column (*i.e.*, position in the seed motif) the best nucleotide element was given value equal to 1 and unacceptable nucleotides (*i.e.*, the other three elements) were given a very small number close to zero. The optimization step aims to find the optimal weight matrix by minimizing an error function:

$$(C,\left\{F_{e\phi }\right\},\left\{w\right\})=\text{argmin}\sum_{e}\sum_{g}{\left(Z_{ge}-F_{e\phi }\sum_{i\in S_{g}}\prod_{j=1}^{L_{\phi }}w_{\phi jb_{i+j-1}}(S_{g})-C\right)}^{2}$$

The indices *ϕ*, *e*, and *g* label RBP, IP experiment replicate, and gene, respectively. Here, *Z* represents the rank-quantile transformed binding data for training set. The training set was obtained by randomly selecting 50% of the data from each transformed IP experiment. The motif seed finding step and subsequent optimization step of the PSAM are both performed on the training set. Once the optimization step converges for this PSAM, the residues of *Z* are then used for the next seed finding and optimization iteration. The intercept *C* represents the genome-wide basal expression level when no preferred motif is present on the sequence. The slope *F* reflects a combination of the activity of the RBP under the media conditions in which binding was assayed, and the efficiency of the protocol for the particular technical replicate. We split every column of the binding data randomly into two sets of equal sized training and test sets and ran MatrixREDUCE on the training set of all experimental replicates of an RBP simultaneously (using command line option -mf). For every RBP, we searched for binding motifs on the whole mRNAs, 5′ UTRs, ORFs and 3′ UTRs sequences separately. For Idh1p, Nrd1p, Tdh3p, and Vts1p, we also ran the software without the -mf argument because for all of these factors one of the experimental duplicates was missing more than 40% of the data points. We obtained the *Saccharomyces cerevisiae* UTR sequences RNA-seq data (Nagalakshmi *et al.* 2008). mRNA ORF sequences were downloaded from Yeast Genome Database (*Saccharomyces* Genome Database; www.yeastgenome.org). For all RBPs, we searched for PSAMs of length 1−10 iteratively with a p-value cut-off of 0.001.

### Computational validation of PSAMs

We calculated the affinity scores of the discovered PSAMs using the AffinityProfile software from the REDUCE Suite package. We then calculated the Pearson t-value and Spearman p-value for the correlation of the affinity scores to the test data set. We further tried to capture low-specificity flanking sequences for PSAMs that passed a validation step on the test set (*i.e.*, the remaining 50% of each IP experiment). We extended the flanking sides at most one nucleotide position (*i.e.*, added column (1,1,1,1) to the flanks of the PSAM) and ran the OptimizePSAM software from the same package using the PSAM with added columns on the sides as seed. We continued adding columns to the sides of the PSAM until no nucleotide’s weight at the added side flanks was less than 0.1. In the case of Nrd1p PSAM optimization we neglected this criterion where the matrix element for G nucleotide at position 8 was equal to 1 × 10^−7^. Further flank addition and optimization of this PSAM resulted in optimization divergence after several rounds. At the end of each optimization cycle, we validated the newly extended PSAM by calculating the Pearson t-value and Spearman p-value on the test set. After this step, we ran OptimizePSAM using the full data on the PSAMs passed validation steps.

We selected the final set of PSAMs by performing a specificity test. For each PSAM, we calculated the Pearson t-values for correlation of each of the 132 IP experiments to the affinity score on the 5′ UTR, ORF, and 3′ UTR separately. We only accepted those RBP/region combinations for which the PSAM affinity score exclusively was correlated to the IP data from which it was derived. In the case of YLL032C, we used the complete mRNA sequence. For YLL032C, no statistically significant motif was obtained when MatrixREDUCE was run using the 5′ UTRs, ORFs, or 3′ UTRs separately. Only the search that was performed using the complete mRNA sequences detected a motif for YLL032C.

### Functional assessment of novel motifs

We used two different approaches to functionally assess the discovered PSAMs: Gene Ontology (GO) enrichment analysis (Ashburner *et al.* 2000) and correlation to 167 stress conditions (Gasch *et al.* 2000). We used GO enrichment scoring analysis to detect the underlying regulatory program, cellular state, or cellular component for the novel motifs. For each GO category, we tested whether the affinity scores for a PSAM on ORF or UTR sequences were associated with a specific biological pathway or not. We applied the nonparametric Wilcoxon-Mann-Whitney test to determine whether the affinity scores of mRNA regions within a particular GO category have a different distribution than the affinity scores for all other mRNAs. We used an iterative procedure for removing the effect of redundant nested GO categories (Boorsma *et al.* 2008). We only considered GO categories with at least 10 genes. To correct for multiple testing, we performed a Bonferroni correction on the resulting p-values accepting only categories with p-values smaller than 0.01/N, where N is the number of unique GO categories.

To perform the GO enrichment analysis, we used packages *GO.db* and *org.Sc.sgd.db* for *Saccharomyces cerevisiae* from the Bioconductor website within the R statistical programming environment (www.Bioconductor.org).

To further validate novel motifs, we correlated the affinity scores of the 25 factors to expression data from 173 different stress conditions. The stress conditions include heat shock, exposure to oxidative or reductive chemical agents, nutrients or amino acid starvation, and changes in osmolarity. We performed least-squares multiple linear regression of the genome-wide mRNA expression levels of each condition to the affinity scores of all of the selected RBP/region combination and compared the t-values of the regression coefficients among different conditions.

### Inferring segregant-specific RBP activities

From the RBP motif discovery analysis we obtained 25 independent RBP/region combinations. As in Lee and Bussemaker (2010), we used the affinity scores of the obtained PSAMs as a predictor for mRNA differential expression (Foat *et al.* 2006; Bussemaker *et al.* 2007). The study by Lee and Bussemaker (2010) showed that the effect of *trans*-acting polymorphisms on mRNA expression via the activity of transcription factors is independent of the effect due to allelic variation in *cis*-regulatory sequences. Because this study focused on the *trans*-acting genetic variation, and also to simplify the analysis, all affinity scores were calculated based on the transcript sequences of S288c, a strain isogenic to BY. We performed genome-wide multiple regression on the 25 RBP/region combinations of every segregant mRNA expression log_2_-ratios to infer segregant-specific activity levels of the RBPs.

$$y_{gs}=\beta_{0s}+\sum_{\phi }\beta_{\phi s}K_{\phi g}$$

Where $y_{gs}$ represents the differential mRNA levels of gene *g* for segregant *s* relative to the reference. Here, the regression coefficient $\beta_{\phi s}$ represents the activity level of RBP *ϕ* for segregant *s*, whereas $K_{\phi g}$ represents the aggregated affinity of the 5′ UTRs, ORFs, 3′ UTRs, or the complete mRNA sequence of gene *g* of the BY strain for the factor under consideration, as mentioned previously.

### aQTL mapping

Significant aQTL regions were discovered by splitting the multiple regression coefficient between BY and RM at every marker and testing for the significance of the difference between the distributions of the two groups of coefficients using the composite interval mapping (CIM) method for maximum resolution (Zeng 1994). CIM uses multiple regression on multiple markers to obtain a precise mapping of the QTL. We used CIM implementation in R/qtl package by (Broman *et al.* 2003). LOD score, an acronym for “logarithm of the odds ratio,” was calculated to check for linkage. We calculated the LOD score to test the linkage of the RBPs inferred activities at each locus. We performed 200 independent random permutations on the columns of expression data (*i.e.*, segregants) while preserving the genotype data to get LOD score thresholds at 1% false discovery rate (FDR) level. We obtained this threshold for each RBP/region combination separately.

To confirm that the detected aQTL regions for the RBPs are modulated by *trans*-acting factors and not dominated by a single-gene eQTL, we repeated the analysis after eliminating 3 groups of genes: (i) genes that encode RBPs; (ii) genes fully or partly located within 10 kb of aQTL markers; and (iii) genes whose expression variation maps to a marker 20 kb of aQTL markers, and scoring an affinity of at least 50% of the maximum for the RBP under consideration (see Figure S5). To find the latter group, we carried out eQTL analysis using the expression of each gene as a trait and calculated LOD score for every marker using CIM method. We combined these three groups of genes and removed them from the affinity and expression data sets for each RBP separately. This way, the activity calculation for each factor was not affected by elimination of unrelated genes.

### Protein−protein interaction data

Protein−protein interaction data were downloaded from (thebiogrid.org) for yeast, April 2012. We used it to detect any known genetic or physical interactions with the genes located in aQTL regions.

### Validation of predicted locus−RBP associations

We used gene expression profiles for two mutant strains growing in glucose medium collected by Smith and Kruglyak (2008) where *IRA2* alleles were swapped between the BY and RM strains. We label the strain carrying the RM allele of *IRA2* in the BY background as (RM@*IRA2*) and the strain carrying the BY allele of *IRA2* in the RM background as (BY@*IRA2*). The reference sample used for the gene expression measurements was pooled parental mRNA (BY and RM). To obtain the net effect of the *IRA2* allele replacement on the genome-wide mRNA levels ($y_{g}$), we subtracted the mean log-ratio of the related background of each mutated strain (shown for RM@*IRA2* strain as an example):

$$y_{g}^{BY\overset{}{\to }RM@IRA2}={\text{log}}_{2}(\frac{\left[{\text{mRNA}}_{g}](RM@IRA2,\text{glucose}\right)}{\left[{\text{mRNA}}_{g}](\text{pool}\right)})-\bar{{\text{log}}_{2}(\frac{\left[{\text{mRNA}}_{g}](BY,\text{glucose}\right)}{\left[{\text{mRNA}}_{g}](\text{pool}\right)})}$$

We performed multiple regression between the aforementioned data vector and the affinity scores of 25 RBP/region combinations. Similarly, we calculated the relative mRNA expression for the RM strain when *IRA2* was replaced by the BY allele in the RM background and carried out multiple regression analysis. To capture the effect of the *IRA2* allele swap between the two backgrounds, we subtracted the regression coefficients between the two cases for all the 25 combinations. We then permuted the two *y* vectors for all genes 1000 times independently to calculate the statistical significance threshold at 1% FDR level (|y|>2.7).

### Linear model analysis of quantitative RT-PCR data

For each of the three technical replicates of the eight strains, we calculated the difference in C_T_ values between *RRS1* (test) and *THI6* (control). Because of the inverse relation between mRNA level and C_T_, we subtracted the test value from the control value. We performed a least-squares fit of the following linear model to estimate the effect of *IRA2*, and *PUF4* on the mRNA level of *RRS1*, as well as the genetic interaction between *IRA2* allelic status and *PUF4* deletion:

$$C_{T}(THI6)-C_{T}(RRS1)=b_{0}+b_{1}x_{RM@IRA2}+b_{2}x_{PUF4\Delta }+b_{3}(x_{RM@IRA2}\times x_{PUF4\Delta })+\varepsilon$$

Here each independent variable takes on the value *x* = 1 when the *IRA2* allele is RM, or the *PUF4* gene is deleted, respectively, and *x* = 0 otherwise. We fit the model separately for two subsets that were selected based on the background strain BY or RM.

## Results

### Data

For motif discovery, we used RNA immunoaffinity purification data that included a total of 132 IP experiments for 45 RBPs from Hogan *et al.* (2008). For the aQTL search, we used parallel genotyping and genome-wide mRNA expression data (collected in rich-media conditions) for 108 segregants from a cross between two haploid yeast strains: BY4716 (BY) and RM11-1a (RM) (Smith and Kruglyak 2008).

### RBP motif discovery

**Figure 1:**
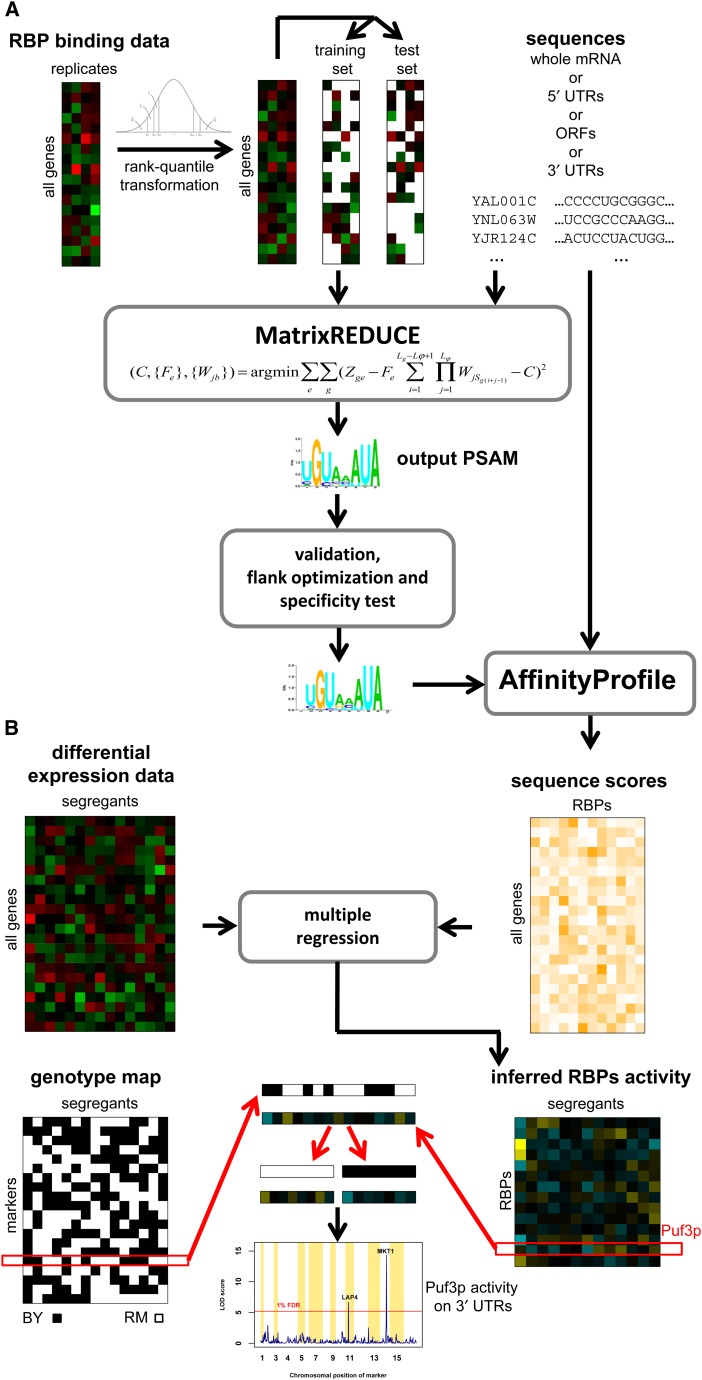
Overview of our computational methodology. (A) Motif discovery. We used MatrixREDUCE on rank-quantile transformed binding data (training set) and mRNA sequences. We repeated this analysis by replacing the complete mRNA sequences by 5′ untranslated regions (UTRs), open reading frames (ORFs), and 3′ UTRs separately. We accepted the position-specific affinity matrices (PSAMs) only if they passed validation on test set and specificity test (exclusive correlation of affinity scores for a RNA-binding protein affinity to its own binding data). (B) Activity quantitative trait loci (aQTL) method. Genome-wide affinity scores were calculated using PSAM and sequences. The affinity scores were used to infer segregant-specific RNA-binding protein (RBP) activities. The activities were obtained by multiple linear regression on differential mRNA expression levels to the affinity scores. The regression coefficients represent the RBP activity levels for each segregant. For linkage analysis, the activities were treated as quantitative traits. For each factor we split the inferred activities of segregants at each marker based on the inherited allele (BY or RM) at that marker. We then test whether the distribution of the activities levels between the two subsets is significantly different using Composite interval mapping. Whenever the distribution of these inferred activity levels of a RBP depends on the genotype variation of a specific chromosomal marker, we obtain a high logarithm of the odds ratio score at that marker, indicating the presence of an aQTL (at 1% false discovery rate level).

Our motif discovery procedure is summarized in Figure 1A and Figure S2. We used the MatrixREDUCE program from the REDUCE Suite software package, which takes as inputs the nucleotide sequences and RBP binding log_2_-ratios for all mRNAs. To reduce the effect of outliers, we applied a quantile-based transformation to the binding data (see the section *Materials and Methods*). To define RNA sequences, we used the annotation from (Nagalakshmi *et al.* 2008) and extracted 5′ and 3′ UTRs, ORFs, and complete mRNA nucleotide sequences. For every RBP, we performed a genome-wide motif search on complete mRNA transcripts, ORFs, 5′ and 3′ UTRs separately. We did this to allow for functional differences within each transcript. In some cases, we also observed that using the complete mRNA sequence hindered our ability to discover motifs. For instance, for Nrd1p and Puf2p, we could only detect statistically significant binding motifs using the 3′ UTRs.

After the training step, using a random sample of 50% of the data, we calculated the affinity scores using the derived position-specific affinity matrices (PSAMs; see the section *Materials and Methods*) and only selected those validated using the remaining 50% of the data. We further optimized these PSAMs by adding flanking sequence (up to one nucleotide on either side) to capture low-specificity bases not identified during the training step. A final optimization step using the complete data set yielded PSAMs for 20 of 45 RBPs (see Table S1 and Table S5).

Regulation of mRNA stability typically is carried out through protein interactions via the 3′ UTR (Grzybowska *et al.* 2001; Mignone *et al.* 2002; Shalgi *et al.* 2005); however, there are exceptions: yeast Khd1p represses *FLO11* expression by binding to its coding region (Wolf *et al.* 2010) and Msl5p binds a specific motif near the intron-exon boundary during splicing (Berglund *et al.* 1997; Garrey *et al.* 2006). To accommodate binding at multiple locations along a transcript, we scored the correlation between 132 IP experiments and the affinity of each mRNA region (*i.e.*, 5′ UTR, ORF, and 3′ UTR) separately. We also required that each PSAM was most correlated with binding for each factor (IP data). Five of the 20 PSAMs we discovered (Idh1p, Mrn1p, Puf1p, Rna15p, and Yra2p) did not pass this specificity test (Figure S4).

**Figure 2:**
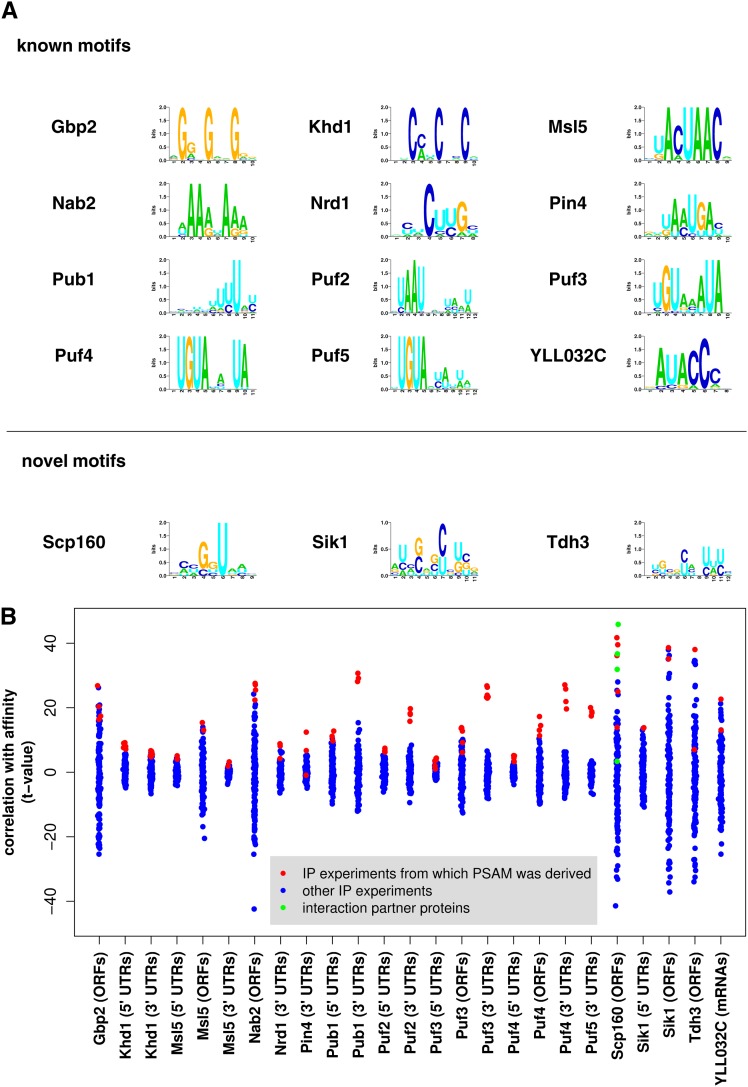
List of selected position-specific affinity matrices (PSAMs) based on the specificity test. (A) List of known and novel RNA-binding protein (RBP) motifs obtained by our motif discovery approach. These 15 PSAMs passed validation and specificity tests. (B) Specificity test. Scatter plot for the factors specificity test where the Pearson t-values of correlation between 132 RBP binding experiments and 25 selected PSAM-region combinations are presented. Only the factors with at least one self RBP immunoprecipitation (IP) experiment t-value (red dots) appearing at the top are shown. The only exception is for Scp160p acting via open reading frames (ORFs) where we have a greater correlation to Bfr1p binding data (green dots). We accepted this PSAM because Scp160p and Bfr1p are known to interact and are coimunoprecipitated in IP measurements.

Figure 2A shows the sequence logos for the final 15 PSAMs, whereas Figure 2B lists the 25 significant RBP/region combinations we detected. There was an exception for Scp160p, for which the aggregated affinity across the ORFs was more correlated to the Bfr1p IP experiment (green dots). This was expected because Bfr1p associates with cytoplasmic mRNP complexes containing Scp160p (Lang *et al.* 2001). There is a large gap between the relevant IP experiments (red dots) and the rest of the IP experiments (blue dots) for the affinity of 3′ UTRs to Pub1p, Puf2p, Puf3p, Puf4p, and Puf5p, indicating that these PSAM are highly specific to the binding data from which they were derived.

### Refined PSAMs for RBPs of known sequence specificity

Of the 15 specific PSAMs we discovered, 12 are broadly consistent with motifs derived previously from the same binding data (Gerber *et al.* 2004; Hogan *et al.* 2008). Our motif for Gbp2p, GRNGNNGR (R is A/G), is predictive for binding in the ORF. Gbp2p is involved in mRNA export from the nucleus to the cytoplasm. The motif HGGUGW (H is A/C/U, W is A/U) previously reported for this protein (Riordan *et al.* 2011) is compatible with our finding. Khd1p is involved in the asymmetric localization of Ash1p in daughter cells, which is a transcription inhibitor of the mating type switch protein encoded by the *HO* gene. Khd1p binds to CNN repeats in coding regions of its targets *in vitro* (Hasegawa *et al.* 2008). A more recent study reported enrichment of YCAY (Y is C/U) in the mRNAs bound to Khd1p (Wolf *et al.* 2010). Msl5p is part of the splicing initiation complex (Abovich and Rosbash 1997) and binds to branch-point sequence UACUAAC (Berglund *et al.* 1997; Garrey *et al.* 2006), in agreement with the PSAM we identified for this protein. Nab2p is involved in poly(A) tail formation control and export of mRNA from nucleus to cytoplasm (Hector *et al.* 2002; Kelly *et al.* 2010). Consistent with our finding, the Nab2p PSAM is enriched with adenine (Kim Guisbert *et al.* 2005). The PSAM we found using Nrd1p binding data has a core motif CUUG. This protein is subunit of the Nrd1p-Nab3p-Sen1p complex, which mediates the termination of small nucleolar RNAs (Vasiljeva *et al.* 2008). It has been reported that Nrd1p binds to GUAR and Nab3p recognizes UCUU or CUUG (Carroll *et al.* 2004; Lunde *et al.* 2011; Porrua *et al.* 2012). Because Nab3p and Nrd1p form a complex, it is not surprising that we identified the Nab3p motif when analyzing the Nrd1p binding data. Indeed, we found a highly similar motif when using Nab3p binding data (p-value < 10^−16^, Spearman rank correlation between aggregated 3′ UTR affinities). The motif we obtained for Pin4p looks similar to the motif reported by (Hogan *et al.* 2008). In the case of Pub1p, a poly(U) binding protein that is essential for stability of many mRNAs (Anderson *et al.* 1993; Matunis *et al.* 1993; Duttagupta *et al.* 2005; Hogan *et al.* 2008; Li *et al.* 2010), our motif is indeed a U-rich element. The YLL032C gene encodes an unannotated protein that may interact with ribosomal complexes (Fleischer *et al.* 2006). Our algorithm found an AUACC motif as reported previously (Hogan *et al.* 2008).

### PUF proteins

Among the RBPs for which we were able to identify a binding motif are four members of PUF family. Not much is known about the physiological role of Puf2p. It interacts preferentially with mRNAs that encode membrane-associated proteins (Gerber *et al.* 2004). In a recent study, it was shown computationally and experimentally that Puf2p binds to a dual UAAU motif with 3 nucleotide linker.(Yosefzon *et al.* 2011). Our PSAM search algorithm captures the same motif. The Puf2p binding consensus motif is distinct from the UGUA-containing motifs bound by Puf3p, Puf4p, and Puf5p (Gerber *et al.* 2004; Foat *et al.* 2005; Miller *et al.* 2008). Puf3p binds nearly exclusively to mRNAs that encode mitochondrial proteins (Gerber *et al.* 2004) and is involved with mitochondrial localization of nuclear-encoded mRNAs (Saint-Georges *et al.* 2008). Puf3p enhances *COX17* mRNA degradation by binding to a UGURNAUA motif in its 3′ UTR (Olivas and Parker 2000; Jackson *et al.* 2004). Puf4p is known to bind to a UGUAUAUUA motif in the 3′ UTR of *HO* endonuclease mRNA and, together with Puf5p, negatively regulates it (Hook *et al.* 2007; Miller *et al.* 2008). Puf4p is also known to bind preferentially to mRNA encode ribosomal proteins (Gerber *et al.* 2004). The PSAMs we found for Puf3p, Puf4p and Puf5p are all in agreement with the motifs reported earlier.

### Novel binding specificities for Scp160p, Sik1p, and Tdh3p

Our method detected novel binding specificities for three RBPs for which previous motif finding attempts had failed. To corroborate these finding, we analyzed the correlation of PSAM-based affinity scores with differential mRNA expression data across 173 different stress conditions (Gasch *et al.* 2000). It was previously shown that this procedure allows us to quantitatively estimate changes in protein-level regulatory activity of RNA-binding *trans*-acting factors (Foat *et al*.). We also performed GO analysis on the *in vitro* affinity scores using the Wilcoxon-Mann-Whitney test (see the section *Materials and Methods*).

Scp160p is an RBP involved in the mating response (Guo *et al.* 2003). It contains multiple heterogeneous nuclear ribonucleoprotein K-homology domains. Scp160 affinity for ORF sequences is highly anticorrelated with expression in YPD stationary phase relative to early log phase, YPD, nitrogen depletion, and heat shock conditions and positively correlated for cold shock and hypo-osmotic shock conditions (see Table S2). GO analysis based on the mRNA affinity scores for Scp160p showed an association with the nitrogen compound metabolic process category (p-value < 10^−8^, Wilcoxon-Mann-Whitney test).

Sik1p (Nop56p) is a component of the box C/D snoRNP complexes that direct 2′-O-methylation of pre-rRNA during its maturation. Our detected motif for Sik1p is enriched in both 5′ UTRs and ORFs of mRNAs. We observed positive correlation of ORF affinity scores with differential expression during YPD stationary phase growth and after heat shock (see Table S2). It could be that Sik1p has a direct or indirect role in rRNA methylation regulation under heat shock and that could explain why we observed positive correlation for the heat shock conditions. GO analysis showed significant association with ribosome and rRNA-related categories (p-value < 10^−9^).

Tdh3p encodes glyceraldehyde-3-phosphate dehydrogenase, which is required during gluconeogenesis and is essential for yeast cells to grow on noncarbohydrate sources such as ethanol and glycerol (McAlister and Holland 1985). The affinity score for Tdh3p correlates positively with expression changes after exposure to menadione, a synthetic nutritional compound, and negatively to sorbitol and nitrogen depletion. GO scoring analysis for this factor showed the categories “intrinsic to membrane,” “thiolester hydrolase activity,” “glucosyltransferase activity,” and “glycerophospholipid metabolic process” to be significantly associated (p-value < 10^−6^ in all cases).

### Dissecting genetic variation in RBP activity across segregants

It has been experimentally validated that by analyzing the mRNA differential expression levels of putative targets of a transcription factor, changes in the protein-level regulatory activity of that factor can be inferred (Boorsma *et al.* 2008). As for the activity levels, it has been shown that they vary among members of a population of an organism and can be treated as a quantitative trait for genetic linkage analysis to capture polymorphisms that modulate the activity of the transcription factors (Lee and Bussemaker 2010). Here we applied a similar approach to identify *trans*-acting loci controlling the activity of RBPs that influence mRNA expression levels through posttranscriptional control of their half-lives (Foat *et al.* 2005).

We combined segregant-specific genome-wide mRNA expression profiles with prior information about the posttranscriptional regulatory network to infer differential RBP activity levels in each of 108 haploid segregants from a genetic cross between a lab (BY) and a wild (RM) strain (Smith and Kruglyak 2008). Figure 1B and Figure S3 illustrate the steps involved in this analysis. We used the 25 RBP/region combinations from the motif discovery analysis to calculate the aggregate affinity scores (Figure 2B). Multiple regression on affinities was then performed independently for each segregant mRNA expression profile, and the regression coefficients were interpreted as estimates of the corresponding RBP activities. We used the CIM method (Zeng 1994) to map aQTLs for each of the 25 RBP/region combinations. To account for multiple testing, we calculated LOD score thresholds corresponding to a 1% FDR by performing 200 permutations (see the section *Materials and Methods*). Table 1 summarizes the results of our analysis. We were able to map at least one aQTL for Khd1p, Msl5p, Pub1p, Puf2p, Puf3p, Puf4p, and the putative regulator YLL032C.

**Table 1:** Discovered aQTL regions for RBPs

RBP	mRNA Region	aQTL Region	Max LOD Score	Direct Interaction	Interaction Type	Reference
Khd1p	3′ UTRs	Chr15	14.6			
		154,310-193,910				
Msl5p	3′ UTRs	Chr15	15.2			
		136,328-170,944				
Pub1p	3′ UTRs	Chr15	6.0			
		154,310-193,910				
Puf2p	5′ UTRs	Chr2	5.8			
		533,269-565,215				
		Chr15	7.0			
		154,310-193,910				
Puf3p	5′ UTRs	Chr2	7.1	*POP7*	GI	(Wilmes *et al.* 2008)
		555,788-592,862				
Puf3p	3′ UTRs	Chr11	6.8	*LAP4*	PI	(Breitkreutz *et al.* 2010)
		229,053-247,943				
		Chr14	13.9	*MKT1*	GI	(Lee *et al.* 2009)
		449,640-502,315				
Puf4p	5′ UTRs	Chr15	10.4			
		154,310-193,910				
Puf4p	ORFs	Chr15	13.5			
		154,310-193,910				
Puf4p	3′ UTRs	Chr15	10.7			
		154,310-193,910				
YLL032C	mRNAs	Chr15	5.9			
		141,634-170,944				

aQTL, activity quantitative trait loci; RBP, RNA-binding protein; LOD, logarithm of the odds ratio; UTR, untranslated region; ORF, open reading frame; GI, genetic interaction; PI, physical interaction.

### Segregation of *trans*-acting alleles decouples Puf3p and Puf4p activity

**Figure 3:**
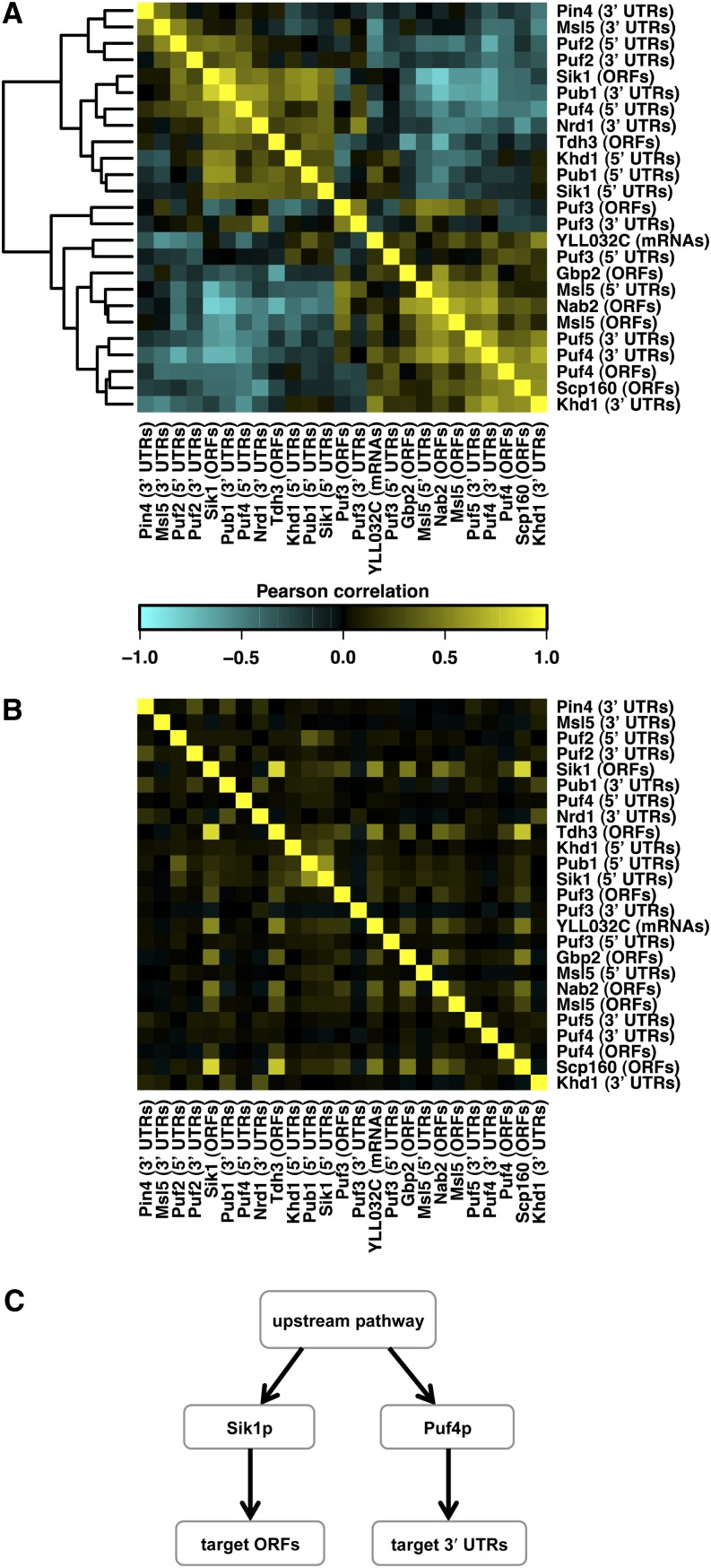
Affinity correlation *vs.* activity correlation. (A) Clustered heatmap of Pearson correlations calculated for the inferred activity levels of 25 factors. The activities were calculated using multiple regression on the genome-wide expression levels to the RNA-binding protein (RBP) affinities on selected mRNA regions. (B) Pearson correlations of affinity scores. For convenience, factors are in the same order as in panel (A). (C) Possible scenario for observing more significant correlations between inferred activity levels of the RBPs. (A) Significant negative correlation between Puf4p activity on 3′ untranslated regions (UTRs) and Sik1p activity on open reading frames (ORFs) (Wilmes *et al.* 2008); However, Puf4p and Sik1p have distinct set of target genes (weak correlation between their affinities as observed in panel B).

The binding specificities of Puf3p and Puf4p differ with respect to the length of the gap between the UGUA and AUA submotifs (*cf*. Figure 2A). As a consequence, they have distinct target sets, as measured in terms of the correlation in binding affinity across all transcripts (Figure 3A). The same observation holds across the larger set of RBPs: with few exceptions, they control independent sets of targets (Figure 3B).

**Figure 4:**
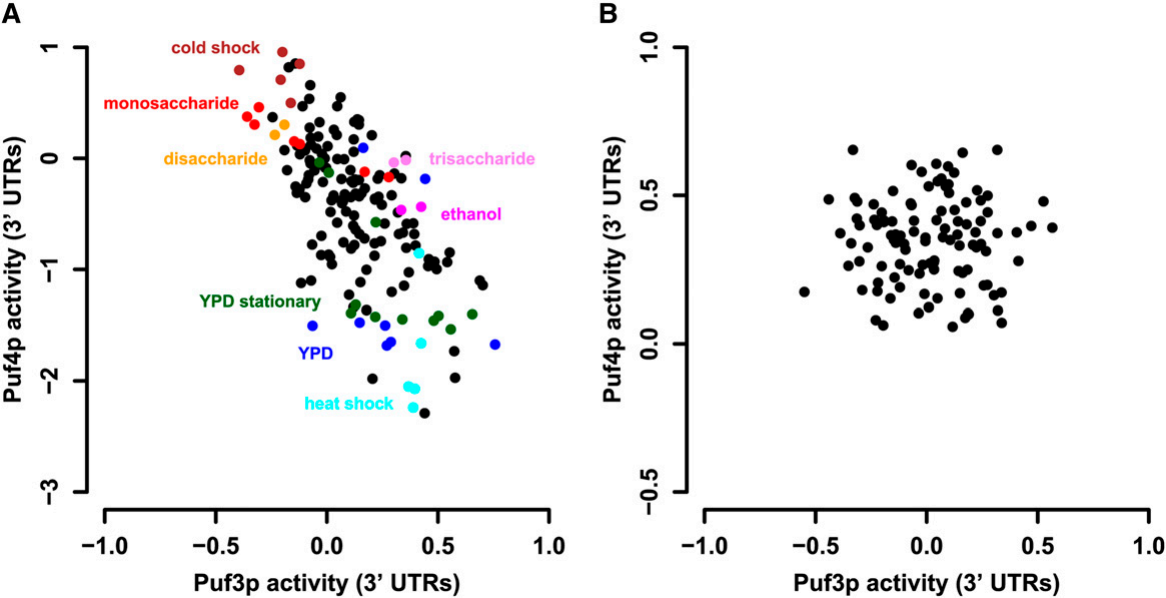
Decoupling of Puf3p and Puf4p activity. (A) Scatter plot of inferred activity levels for (Gasch *et al.* 2000) stress data sets shown for Puf3p [3′ untranslated regions (UTRs)] and Puf4 (3′ UTRs). To infer RNA-binding activities, we performed a multiple regression on the mRNA levels of each experimental condition to all 25 factors affinity scores. (B) Scatter plot of inferred activity among 108 segregants for the same two factors.

It has been previously noted that Puf3p and Puf4p activity levels respond oppositely when cells are exposed to different sugar sources (Foat *et al.* 2005). Consistently, when we analyzed the activity variation for both factors form genome-wide expression data across a variety of stress conditions (Gasch *et al.* 2000), we observed a marked negative correlation (Figure 4A; r = −0.67, p-value < 10^−16^). By contrast, inferred Puf3p and Puf4p activity levels do not correlate across the segregants from the BY-RM cross (Figure 4B; r = −0.001, p-value = 0.99). This suggests that their respective activities are modulated by distinct genetic loci. Thus, our systems genetics approach provides us with a unique opportunity to dissect the connectivity between the upstream TOR signaling pathway and these two factors.

### Recovering MKT1 as an aQTL for Puf3p acting via the 3′ UTR

**Figure 5:**
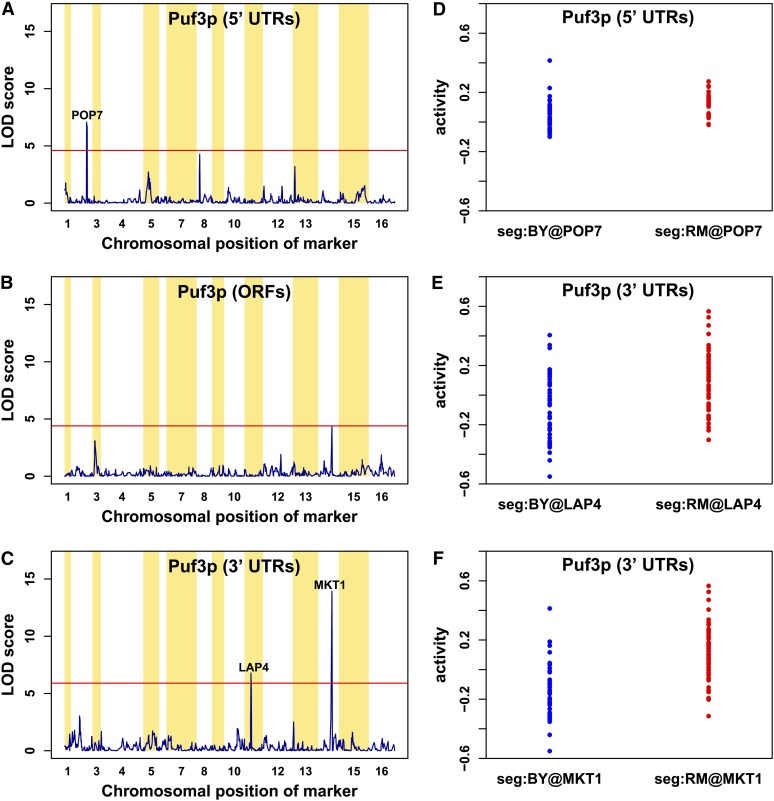
Activity quantitative trait loci (aQTL) profiles for Puf3p. Results for the *trans*-acting genetic modulators of Puf3p activity, mapped using our aQTL method. The significant thresholds at 1% false discovery rate level are calculated using 200 independent permutations of the expression data among segregants (red horizontal lines). We obtained distinct aQTL profiles for Puf3p when using affinity scores on (A) the 5′ untranslated regions (UTRs), (B) open reading frames (ORFs), and (C) the 3′ UTRs. Significant aQTL peaks remained after filtering out for the three groups of genes mentioned previously. We identified *POP7* as a putative modulator of Puf3p activity levels when inferred from the 5′ UTRs for the locus on chromosome II (A). The corresponding split of the activity levels at this marker is shown (D). We detected two possible modulators, *LAP4* on chromosome XI and *MKT1* on chromosome XIV, for Puf3p activity levels when inferred from the 3′ UTRs (C). (E) and (F) present the activity level splits at these two loci.

The LOD score profile shown in Figure 5 highlights the genomic locations at which allelic variation drives variation in Puf3p activity. When we used 3′ UTR sequences (Figure 5, C and F), our method recovered an aQTL on chromosome XIV. This locus was previously discovered computationally and experimentally (Lee *et al.* 2009). The authors suggested that the *MKT1* gene at this locus regulates p-body abundance, which in turn regulates Puf3p target abundance; they also tested the effect of *MKT1* deletion on Puf3p target mRNA expression in a RM background. The genome-wide mRNA expression profile of the MKT1Δ strain was used to demonstrate that Puf3p targets are significantly down-regulated. The Mkt1 protein contains two amino acid polymorphisms between the RM and BY strands: G30D and R453K (Lee *et al.* 2009).

Besides *MKT1*, we identified a second aQTL for Puf3p activity. This locus, which is marginally significant, contains the *LAP4* gene on chromosome XI (Figure 5, C and E). It has been reported that Lap4p physically interacts with Puf3p (Breitkreutz *et al.* 2010) and contains four coding polymorphism between RM and S288c, a strain isogenic to BY.

### A distinct aQTL modulates Puf3p acting via the 5′ UTR

Puf3p is believed to interact with the 3′ UTRs of its targets (Olivas and Parker 2000; Gerber *et al.* 2004; Jackson *et al.* 2004). No evidence of functional interaction with the 5′ UTR has been reported to our knowledge. As mentioned previously, Puf3p activity is modulated by an aQTL at the *MKT1* locus on chromosome XIV when acting through 3′ UTRs of its targets. However, as described above, when we analyzed the binding data for Puf3p, we found that binding motif matches in the 5′ UTR were also predictive of transcript binding. Surprisingly, we found that the activity of Puf3p when acting through the 5′ UTR is modulated by a locus on chromosome II that is distinct from the *MKT1* locus (Figure 5, A and D). This aQTL region contains *POP7*, a gene that is reported to have positive genetic interaction with Puf3p (Wilmes *et al.* 2008). Sequence alignment between the RM and S288c strains revealed a coding polymorphism at amino acid position 58 on the Pop7p sequence: the histidine (H) in the RM strain is a glutamine (Q) in S288c. Pop7p is the subunit of both RNase MRP and nuclear RNase P; RNase mitochondrial RNA processing cleaves pre-rRNA, whereas nuclear RNase P cleaves tRNA precursors to generate mature 5′ ends and facilitates turnover of nuclear RNAs (Chamberlain *et al.* 1998; Houser-Scott *et al.* 2002). This makes the H58Q polymorphism a prime candidate for experimental validation. The same aQTL region also contains two mitochondrial related genes *EHT1* and *FZO1*. The coding region of these genes contains three coding and three noncoding polymorphisms for the former and two coding and ten noncoding polymorphisms for the latter. Regardless of the identification of the precise causal single-nucleotide polymorphisms at this locus, our results suggest that Puf3p acts by distinct mechanisms depending on where it binds within the transcript, and responds to distinct upstream pathways.

### Genetic modulation of Puf4p activity

**Figure 6:**
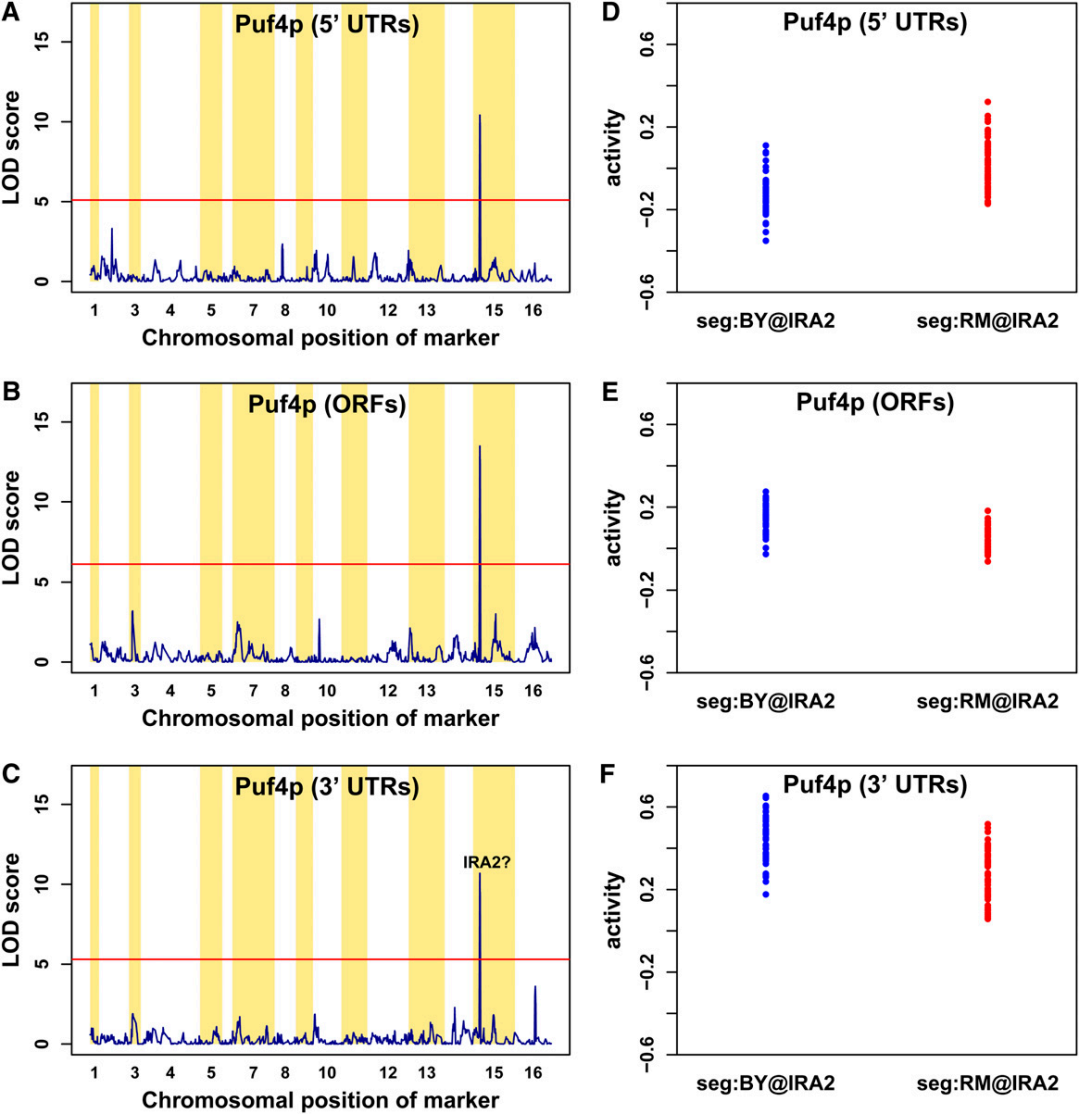
Activity quantitative trait loci (aQTL) profiles for Puf4p. Results of the *trans*-acting genetic modulators of Puf4p activity levels mapped using our aQTL method. The significant thresholds at 1% false discovery rate level were calculated using 200 independent permutations of the expression data (horizontal red lines). The peaks on chromosome XV remained after filtering out for the 3 groups of genes mentioned in the text. (A−C) aQTL profiles for Puf4p activity inferred from 5′ untranslated regions (UTRs), open reading frames (ORFs), and 3′ UTR affinity scores, respectively. Puf4p activity showed a significant linkage to a locus on chromosome XV irrespective of the mRNA region used for affinity calculation. This locus includes the *IRA2* gene. The effect of allelic variation at the *IRA2* locus on Puf4p activity changes direction depending on whether Puf4p acts via 5′ UTRs or 3′ UTRs (D, F).

The aQTL LOD score profile for Puf4p is shown in Figure 6. In this case, regulatory activity seems to be modulated by the same locus on chromosome XV regardless of whether it acts through the 5′ UTR, ORF, or 3′ UTR of its target mRNAs. Strikingly, however, the direction of the change in Puf4p activity from the BY and RM allele at this locus takes opposite values depending on whether Puf4p acts via the 3′ or the 5′ UTR (Figure 6, D and F). Messenger RNA eQTL analysis of mRNA levels of the *PUF4* gene revealed a linkage to the same locus on chromosome XV. This finding suggests that the detected difference in the activity of Puf4p between BY and RM is due to transcriptional and/or posttranscriptional variation between the two strains. One of the genes located in the aQTL region is *IRA2*, which encodes a GTPase-activating protein that negatively regulates Ras signaling and controls intercellular cAMP levels (Tanaka *et al.* 1990). Puf4p interacts with Tpk1p, cAMP-dependent protein kinase catalytic subunit (Cannon and Tatchell 1987; Toda *et al.* 1987; Ptacek *et al.* 2005). Indeed, the genome-wide expression profiles associated with allele replacement from BY@*IRA2* to RM@*IRA2* (Smith and Kruglyak 2008) correlated significantly with Puf4p affinity scored on the 3′ UTR (r = +0.10; t = +5.6; p-value = 2.03 × 10^−8^; see the section *Materials and Methods* for details).

The expression response to *IRA2* allele replacement did not correlate with affinity scored on the ORF or 5′ UTR. This finding suggests that a polymorphism that is genetically linked to but outside *IRA2* is responsible for modulation Puf4p activity as it acts via the 5′ UTR. Possible causal genes are *REX4* and *BRX1*, both of which have putative roles in pre-rRNA possessing and ribosome assembly. Puf4p is known to interact with mRNAs encoding nucleolar rRNA-processing factors (van Hoof *et al.* 2000; Kaser *et al.* 2001; Eppens *et al.* 2002). The coding region of *REX4* contains three coding single-nucleotide polymorphisms between the RM and S288c strains: the asparagine (N) at position 34, phenylalanine (F) at position 155, and lysine (K) at position 248 in RM are lysine (K), leucine (L), and arginine (R) in S288c, respectively. There is a single noncoding polymorphism at position 243 within the coding region of *BRX1*; the thymine in RM is cytosine in S288c.

The activities of five other RBPs (Khd1p, Msl5p, Pub1p, Puf2p, and YLL032C) are also linked to the *IRA2* locus. Using the *IRA2* allele replacement data again to test these aQTL associations, we found that only the affinity of Puf2p via the 5′ UTR (r = –0.079; t = –4.4; p-value = 1.05 × 10^−5^) was significantly correlated with differential mRNA expression. Even though we found the linkage to *IRA2* locus and significant correlation between *IRA2* allele replacement data and the affinity score of Puf2p, no evidence has been reported for a connection between them in the literature thus far.

### Validation of detected loci using *IRA2* allele replacement

**Figure 7:**
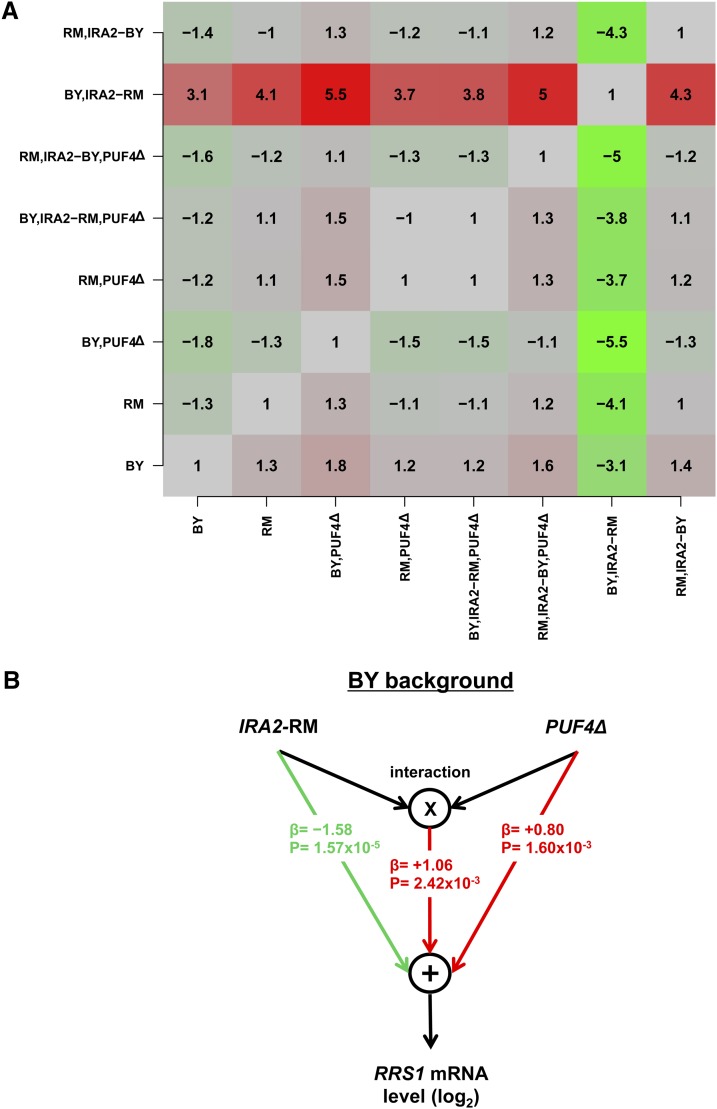
RT-PCR results for *RRS1* expression. (A) Heatmap representation of fold-difference in (normalized) expression for all pairwise combinations of the eight strains used. The fold changes represent the change of RRS1 expression from rows to columns. Red, green, and gray represent increase, decrease, and no change in the expression level, respectively. (B) Graphical representation of the effect on the expression of *RRS1* by *IRA2*-RM and deletion of *PUF4*. Here, β and P represent the regression coefficients and their p-values from the linear model on the *RRS1* quantitative RT-PCR data (see the section *Materials and Methods*).

To test our computational prediction that the activity of Puf4p is influenced by the allelic variation at the *IRA2* locus, we used RT-PCR to monitor expression of *RRS1*, a representative target of Puf4p. To normalize our *RRS1* measurements, we used as a nontarget control *THI6*, based on the criteria that it did not show any notable predicted binding affinity for Puf4p according to our PSAM, was not enriched for Puf4p binding in the study by (Hogan *et al.* 2008), and showed no significant expression difference after IRA2 allele replacement (Smith and Kruglyak 2008). We carried out three technical replicates for each strain (see Figure S6 and Table S3). When active, Puf4p destabilizes its target mRNAs through interaction mostly via 3′ UTR of its targets (Hook *et al.* 2007; Miller and Olivas 2011). Indeed, we observed that the expression of *RRS1* increases 1.8 fold when *PUF4* is deleted in the BY background (Figure 7). The expression of *RRS1* in the same BY background decreased by 3.1-fold when *IRA2* was replaced with the RM allele, indicating that Puf4p was more active. This is consistent with the prediction by our aQTL analysis that Puf4p activity is modulated by the *IRA2* locus (Figure 6, C and F). Since our aQTL analysis treats each locus as independent, we performed the same analysis in the RM background (Figure 7). Surprisingly, we did not observe any change in *RRS1* expression either upon deletion of *PUF4* or allele replacement for *IRA2*. This suggests that in the RM background *RRS1* becomes insensitive to Puf4p activity.

To analyze the results from the quantitative RT-PCR, we performed a least-squares fit of a linear model to the difference in normalized mRNA expression level (see the section *Materials and Methods*). Performing the regression on the four strains with BY background, we obtained an excellent fit (R^2^ = 0.96), with a significant negative effect of allele replacement to RM@IRA2 (t-value = –9.2; p-value = 1.6 × 10^−5^), a significant positive effect of *PUF4* deletion (t-value = +4.7; p-value = 1.6 × 10^−3^), and a significant positive interaction between these two predictors (t-value = +4.4; p-value = 2.4 × 10^−3^). The signs of the regression terms are all consistent with our aQTL-based findings and the known role of PUF4 as a destabilizer of mRNA transcripts (Hook *et al.* 2007; Miller and Olivas 2011). In addition, once Puf4p is absent the allelic identity of *IRA2* does not have any significant effect on *RRS1* based on the positive sign of the interaction term. The fit parameters of the regression on the 4 strains with RM background were all insignificant. It could be that the effects are less severe on this particular target. Taken together, these results validate our aQTL prediction for *IRA2*-Puf4p, but also point to additional genetic complexity that remains to be elucidated.

## Discussion

We have presented a method for identifying *trans*-acting genetic modulators of gene expression, which uses mRNA expression and genotyping data from a segregating population. We used this method to detect aQTL of RBPs. The activities are inferred from RBP binding preferences and the expression data. The inferred activity levels of the RBPs are treated as quantitative traits and were mapped to the chromosomal marker using genotype data. Our method aims to identify posttranscriptional regulatory mechanism underlying genetic variation in gene expression levels.

We applied our aQTL method to a data set for 108 segregants from a genetic cross between two yeast strains (Smith and Kruglyak 2008). RBP sequence specificities were obtained by our motif discovery approach. We calculated the affinity scores for the 25 RBP/region combinations and detected 12 locus-RBP linkages of which only one was previously reported. We recovered the *MKT1* locus on chromosome XIV as a putative modulator of Puf3p activity inferred from 3′ UTRs (Lee *et al.* 2009). Interestingly, we found different loci as modulators of Puf3p when using the 5′ and 3′ UTRs. We also predicted and experimentally validated *IRA2* as a possible modulator of Puf4p activity when the 3′ UTRs affinities were used to infer the activities. Allelic variation at the *IRA2* locus has been shown to be an important determinant of phenotypic differences between the BY and RM strains (Smith and Kruglyak 2008; Chen *et al.* 2009; Litvin *et al.* 2009; Lee and Bussemaker 2010). Taken together, these results show that post-transcriptional regulation accounts for at least some of these differences.

Our motif discovery approach is based on biophysical modeling of the binding of RBPs to target RNAs. It detects potential regulatory elements within RNA sequences that are recognized by diverse RBPs. Our algorithm searches for binding sites in the form of position-specific affinity matrices (PSAMs). Most approaches either impose a threshold to filter RBPs binding data or use gene expression data in combination with mRNA half-lives to identify stability motifs associated with RBPs. Measuring mRNA half-lives requires transcription arrest, which can interfere with the post-transcriptional control of mRNAs under study (Grigull *et al.* 2004). Hence, the interpretation and usage of mRNA half-lives should be performed cautiously. By contrast, our model is not based on defining a target set or mRNA half-lives.

The biophysical model that underlies our method assumes that binding of RBP to mRNA transcripts occurs at nonsaturating concentrations. This indeed seems to be a reasonable assumption. For example, the total number of Puf3p and Puf4p proteins in haploid S288c cells was found to be 846 and 721 molecules, respectively, and therefore the total protein concentration for these factors is ~30 nM (Ghaemmaghami *et al.* 2003). The dissociation constant for the optimal binding sequence for Puf3p equals ~3 nM (Zhu *et al.* 2009) and that for Puf4p ~14 nM (Miller *et al.* 2008). Considering that the free protein concentration is likely to be much smaller than the total protein concentration, and that the dissociation constant increases for suboptimal binding sites, we believe that our assumption of lack of binding saturation is valid.

The PSAMs discovered for 12 RBPs agree with previously reported consensus motifs in other studies. In addition, we discovered three novel motifs for Scp160p, Sik1p, and Tdh3p. The functional validation results from GO enrichment analysis and condition-specific genome-wide mRNA expression data suggest that these novel motifs could be the binding site for Scp160p, Sik1p and Tdh3p or their cofactors. Since we used binding data obtained using imunoaffinity purification, the pulled-down mRNA molecules could plausibly be bound indirectly by the RBP, in which case the motifs obtained would reflect the RNA binding specificity of the cofactor(s). Experimental follow up will be required to rule out this possibility and further validate our new findings.

Taken together, our findings highlight the importance of posttranscriptional regulation that reflects in the mRNA stability by RBPs. Our approach is not yeast-specific and can be applied to other organisms.

## Supplementary Material

Supporting Information
