# Supplementary material for: Harnessing Natural Sequence Variation to Dissect Posttranscriptional Regulatory Networks in Yeast
Source: G3 (Bethesda). 2014 Jun 17;4(8):1539–53. doi: 10.1534/g3.114.012039 (PMC4132183; doi:10.1534/g3.114.012039)
Supplement: Supporting Information [file supp_4_8_1539__index.html]

Harnessing Natural Sequence Variation to Dissect Posttranscriptional Regulatory Networks in Yeast — Supporting Information 

# Harnessing Natural Sequence Variation to Dissect Posttranscriptional Regulatory Networks in Yeast

## Supporting Information for Fazlollahi *et al.*, 2014

**Files in this Data Supplement:**

- Supporting Information - Figures S1-S6, Tables S1-S5, and Supporting Information References (PDF, 412 KB)
- Figure S1 - Schematic representation of the rank-quantile transformation step. (PDF, 143 KB)
- Figure S2 - The flowchart representation of our motif search approach. (PDF, 141 KB)
- Figure S3 - The flowchart representation of our aQTL analysis. (PDF, 141 KB)
- Figure S4 - Specificity test of all significant PSAMs derived for the RBPs. (PDF, 198 KB)
- Figure S5 - aQTL results for all of the 25 accepted RBP/feature combinations. (PDF, 131 KB)
- Figure S6 - Barplot showing the CT values for the qRT-PCR measurements of *RRS1* (test) and *THI6* (control) expression level. (PDF, 140 KB)
- Table S1 - PSAMs statistics on full data from optimization step (significant PSAMs). (PDF, 135 KB)
- Table S4 - List of genotype of strains used in RT-PCR. (PDF, 134 KB)
- Table S5 - Motifs obtained by MatrixREDUCE and reported in the literature for 15 RBPs. (PDF, 267 KB)
- Table S2 - The correlation results for the 25 RBP/regions combinations and the stress condition data. (.xlsx, 106 KB)
- Table S3 - Mean *CT*, raw and normalized fold change results for *RRS1* expression by RT-PCR experiment. (.xlsx, 53 KB)
