# Supplementary material for: Harnessing Natural Sequence Variation to Dissect Posttranscriptional Regulatory Networks in Yeast
Source: G3 (Bethesda). 2014 Jun 17;4(8):1539–53. doi: 10.1534/g3.114.012039 (PMC4132183; doi:10.1534/g3.114.012039)
Supplement: Supporting Information [file supp_g3.114.012039_012039SI.pdf]

G3·Genes | Genomes | Genetics

Harnessing natural sequence variation to dissect post-transcriptional regulatory networks in yeast

Mina Fazlollahi [*,§], Eunjee Lee [†], Ivor Muroff [*], Xiang-Jun Lu [*,§],
Pilar Gomez-Alcala [§,‡], Helen C. Causton [*,2]
and Harmen J. Bussemaker [*,§,1]

[*]Department of Biological Sciences, Columbia University, New York, NY 10027
[§]Department of Systems Biology, Columbia University, New York, NY 10032
[†]Genetics and Genomic Sciences, Mount Sinai Hospital, New York, NY 10029
[‡]Electrical Engineering Department, Columbia University, New York, NY 10027

Corresponding authors:
[1] 607 Fairchild Bldg, 1212 Amsterdam Ave, New York, NY 10027, Tel: 212-854-9932, email: hjb2004@columbia.edu
[2] 607 Fairchild Bldg, 1212 Amsterdam Ave, New York, NY 10027, Tel: 646-894-7260, email: hc2415@columbia.edu

DOI: 10.1534/g3.114.012039

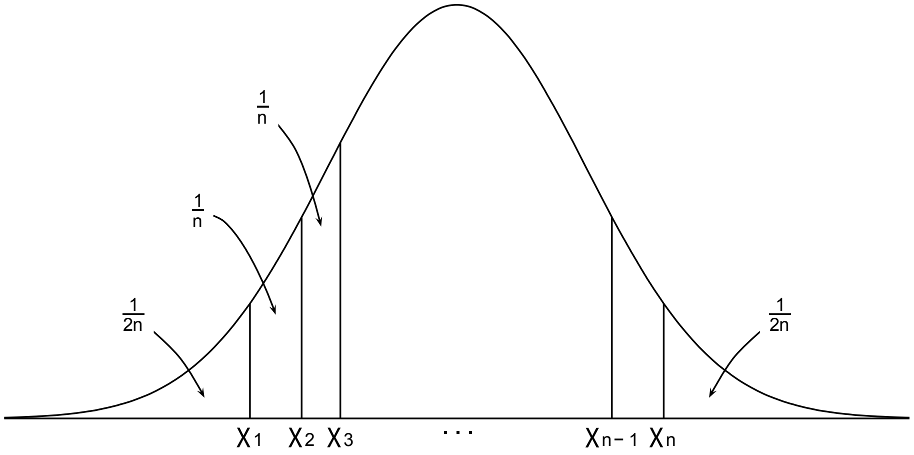

Figure S1 Schematic representation of the rank-quantile transformation step. The transformation is applied to each column (size n) of the binding data. We assigned i^{th}-quantile value (χ_i) to the ith element based on the rank of data point x_i.

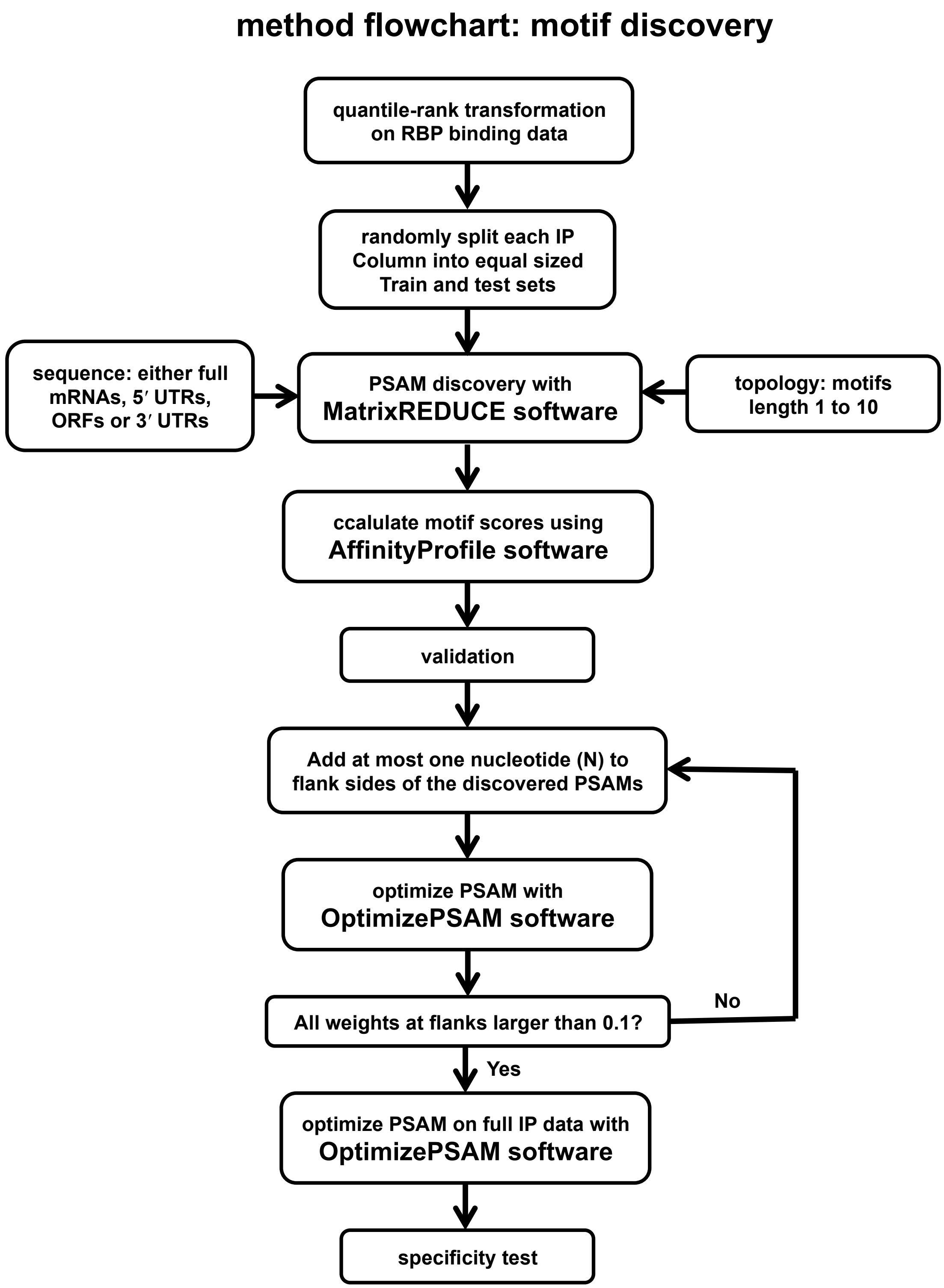

Figure S2 The flowchart representation of our motif search approach.

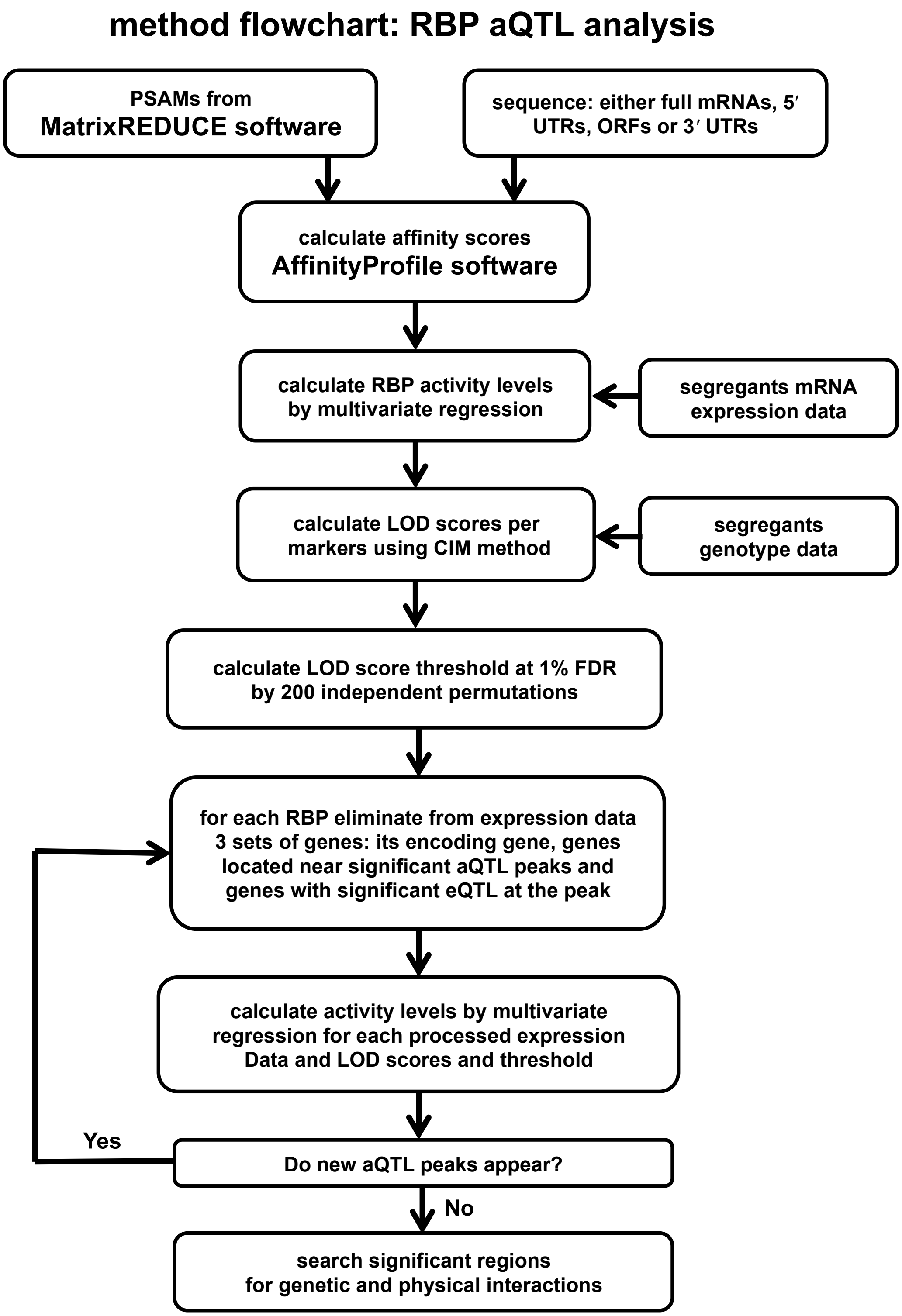

Figure S3 The flowchart representation of our aQTL analysis.

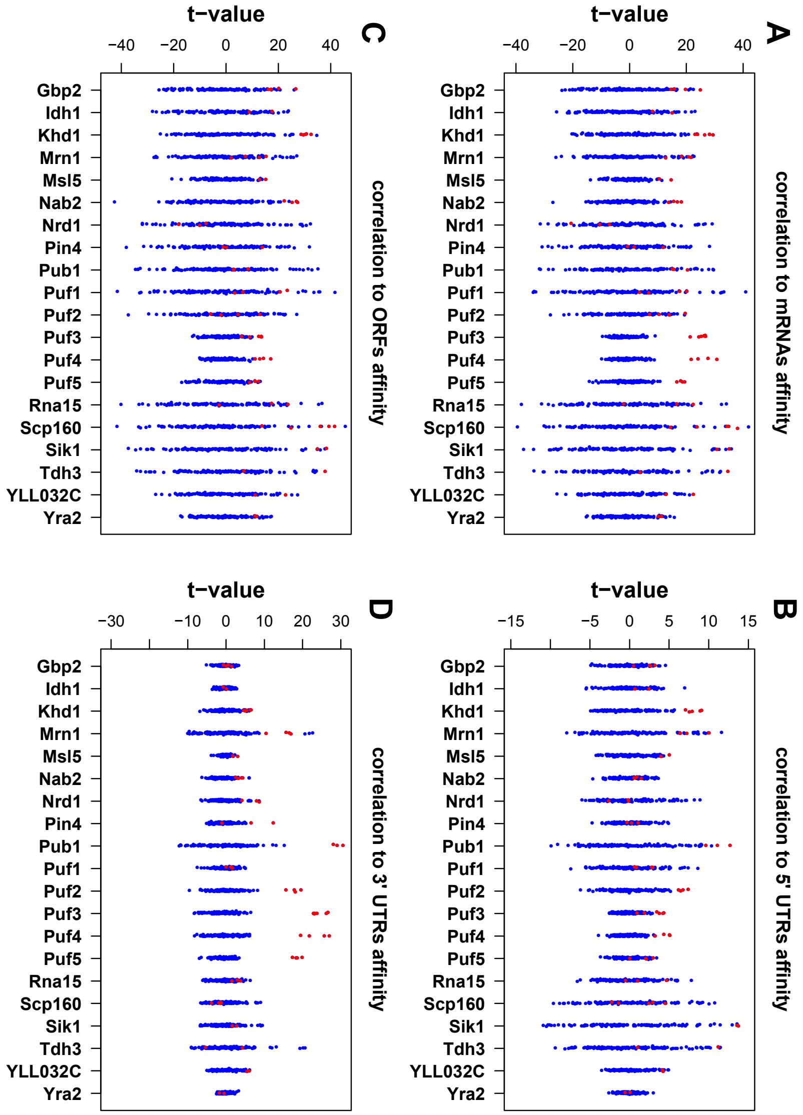

Figure S4 Specificity test of all significant PSAMs derived for the RBPs. The scatter plots display the correlation of the RBP binding data to the affinity scores of the 20 PSAMs calculated on (A) complete mRNA sequence, (B) 5' UTRs, (C) ORFs, and (D) 3' UTRs.

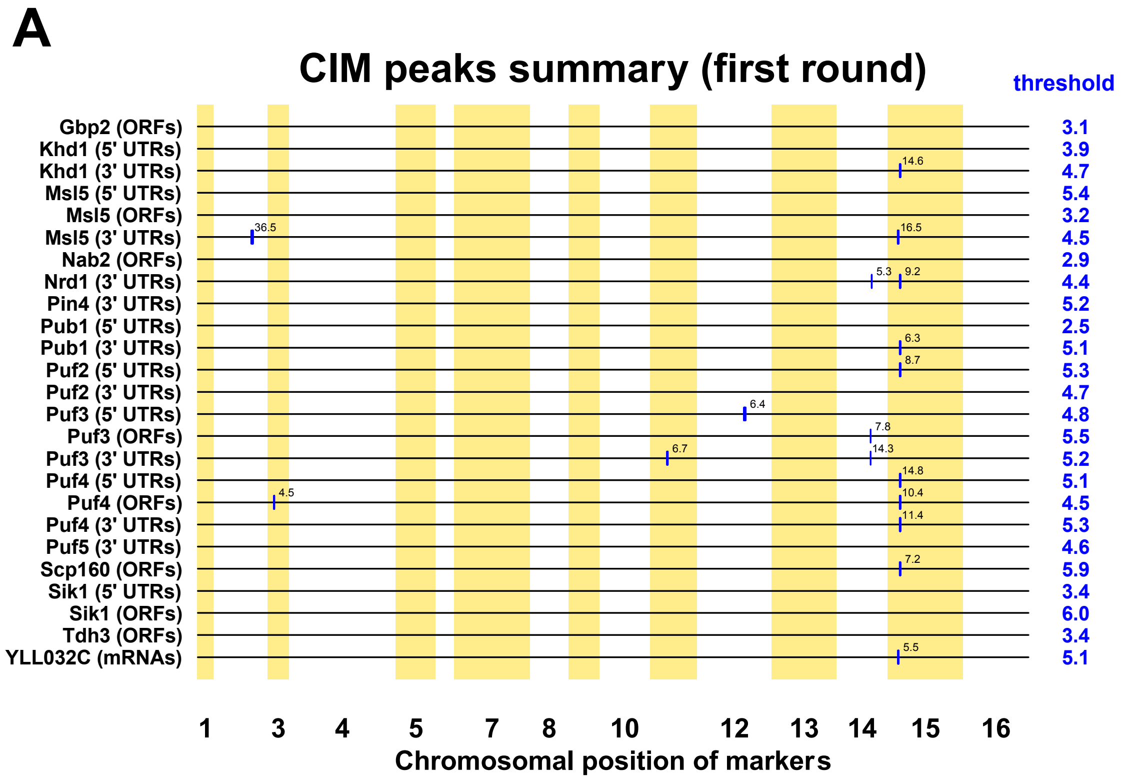

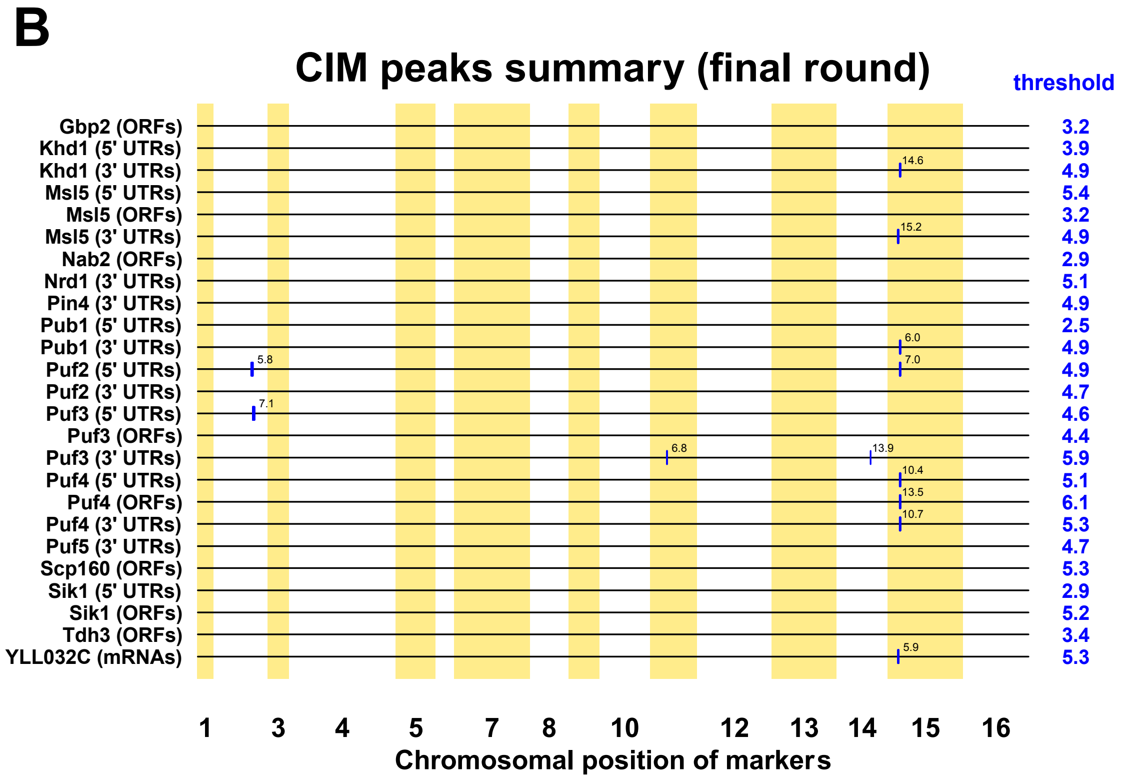

Figure S5 aQTL results for all of the 25 accepted RBP/feature combinations. (A) shows the significant peaks obtained by composite internal mapping (CIM) method when all genes were included (first round), and (B) shows the results after eliminating neighboring genes for each peak, genes with significant eQTL at these peaks and genes encoding the RBPs (last round). After 3 round of gene elimination, no new peak appeared.

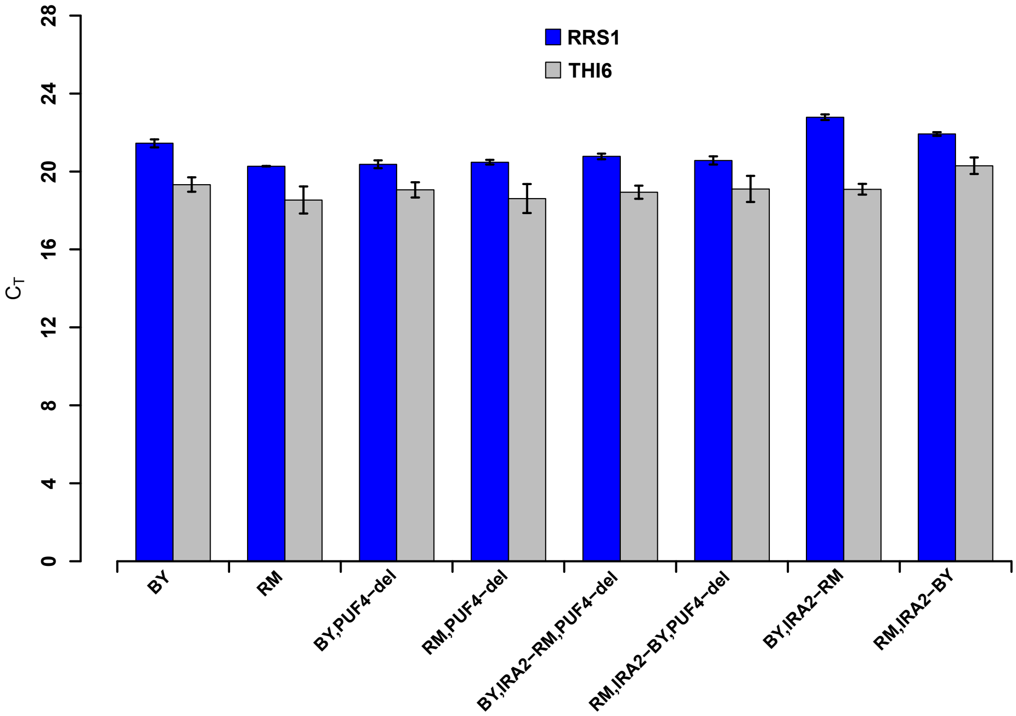

Figure S6 Barplot showing the C_T values for the qRT-PCR measurements of *RRS1* (test) and *THI6* (control) expression level. The mean and standard deviation are calculated based on the 3 technical replicates for each strain.

Table S1 PSAMs statistics on full data from optimization step (significant PSAMs)

RBP	mRNA region[a]	Pearson t-values on IP experiments	Spearman p-values on IP experiments	Accept[b]
Gbp2p	ORFs	25.3, 19.5, 15.8, 14.6	$1.2e^{-133}$, $3.1e^{-105}$, $1.9e^{-31}$, $4.0e^{-27}$	Yes
Idh1p	ORFs	9.1, 15.8	$2.7e^{-27}$, $1.3e^{-86}$	No
Khd1p	ORFs	27.2, 25.7, 28.5, 23.1, 22.2	$3.0e^{-89}$, $2.0e^{-74}$, $2.0e^{-109}$, $3.6e^{-68,}$ $1.0e^{-50}$	Yes
Mrn1p	mRNAs	21.8, 21.0, 18.5, 12.9	$1.3e^{-83}$, $3.0e^{-68}$, $1.8e^{-71}$, $8.4e^{-19}$	No
Msl5p	ORFs	9.3, 13.8	0.32, $1.8e^{-11}$	Yes
Nab2p	ORFs	23.8, 22.4, 21.3, 18.8	$1.7e^{-142}$, $1.5e^{-114}$, $3.3e^{-122}$, $3.4e^{-114}$	Yes
Nrd1p	3′ UTRs	4.2, 7.7, 8.4	$3.0e^{-8}$, $1.6e^{-13}$, $3.1e^{-16}$	Yes
Pin4p	3′ UTRs	6.3, -1.0, 11.5	$1.5e^{-7}$, $1.9e^{-4}$, $4.0e^{-15}$	Yes
Pub1p	3′ UTRs	28.3, 27.2, 30.0	$8.7e^{-143}$, $1.6e^{-145}$, $2.8e^{-152}$	Yes
Puf1p	ORFs	17.9, 3.3, 6.9, 20.3	$2.3e^{-65}$, $7.0e^{-25}$, $2.1e^{-19}$, $1.8e^{-89}$	No
Puf2p	3′ UTRs	15.6, 18.2, 17.6, 16.5	$9.0e^{-12}$, $2.4e^{-27}$, $6.8e^{-15}$, $1.3e^{-21}$	Yes
Puf3p	3′ UTRs	22.1, 22.8, 21.7, 25.0, 23.2	$1.1e^{-24}$, $1.6e^{-17}$, $2.2e^{-19}$, $8.0e^{-26}$, $1.6e^{-39}$	Yes
Puf4p	mRNAs	21.9, 30.9, 27.8, 24.3	$2.3e^{-73}$, $1.7e^{-123}$, $2.1e^{-73}$, $1.7e^{-43}$	Yes
Puf5p	mRNAs	19.6, 16.8, 18.2, 18.9	$4.6e^{-67}$, $1.0e^{-37}$, $3.5e^{-30}$, $3.5e^{-38}$	Yes
Rna15p	ORFs	16.6, 22.1, -2.4	$3.1e^{-89}$, $1.5e^{-136}$, $1.4e^{-5}$	No
Scp160p	ORFs	15.2, 24.7, 35.9, 38.7, 35.1	$1.9e^{-72}$, $4.4e^{-166}$, $4.4e^{-290}$, 0, 0	Yes
Sik1p	5′ UTRs	13.7, 13.9	$1.4e^{-49}$, $9.1e^{-62}$	Yes
Tdh3p	ORFs	34.0, 5.6	$9.0e^{-272}$, $7.9e\text{-}^{16}$	Yes
YLL032C	mRNAs	22.7, 13.0	$3.7e^{-112}$, $6.1e^{-19}$	Yes
Yra2p	mRNAs	11.4, 10.3	$2.4e^{-14}$, $1.9e^{-11}$	No

[a] represents the region (complete mRNA, 5′ UTR, ORF, or 3′ UTR) the PSAM was trained on

[b] acceptance based on the specificity test

Tables S2-S3
Available for download as Excel files at http://www.g3journal.org/lookup/suppl/doi:10.1534/g3.114.012039/-/DC1

Table S2 The correlation results for the 25 RBP/regions combinations and the stress condition data. This table contains the t-values associated with the regression coefficient for the fitting of affinity scores to the expression data.

Table S3 Mean C_T, raw and normalized fold change results for *RRS1* expression by RT-PCR experiment. The normalization is done based on simultaneous measurements of the *THI6* expression levels.

Table S4 List of genotype of strains used in RT-PCR.

Strain	Parent	Background	Genotype	Reference
BY4716	BY	S288c	*MATα lys2Δ0*	(BRACHMANN *et al.* 1998)
RM11-1a	RM11-1a	RM11-1a	*MATa leu2Δ0 ura3Δ0 ho::KanMX*	(BREM *et al.* 2002)
	BY4741	S288c	*MATa his3Δ1 leu2Δ0 met15Δ0 ura3Δ PUF4::KanMX*	Research Genetics Deletion Collection
IMY204	BY4716	S288c	*MATα lys2Δ0 PUF4::KanMX*	This Study
YLK807/HCY475	BY4724	S288c	*MATa lys2Δ0 ura3Δ0 IRA2RM*	(SMITH and KRUGLYAK 2008)
IMY207	YLK807/ HCY475	S288c	*MATa lys2Δ0 ura3Δ0 IRA2RM PUF4::KanMX*	This study
IMY224	RM11-1a	RM11-1a	*MATa leu2Δ0 ura3Δ0 ho::NatMX*	This study
IMY229	IMY224	RM11-1a	*MATa leu2Δ0 ura3Δ0 ho::NatMX PUF4::KanMX*	This study
YLK810/IMY300	RM11-1a	RM11-1a	*MATa leu2Δ0 ura3Δ0 ho::KanMX IRA2BY*	(SMITH and KRUGLYAK 2008)
IMY236	IMY220	RM11-1a	*MATa leu2Δ0 ura3Δ0 ho::NatMX IRA2BY PUF4::KanMX*	This study

Table S5 Motifs obtained by MatrixREDUCE and reported in the literature for 15 RBPs.

RBP	MatrixREDUCE motif	Reported motif in literature	References
Gbp2	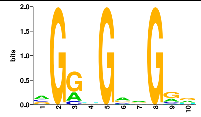	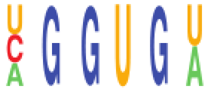	(RIORDAN *et al.* 2011)
Khd1	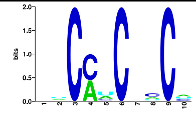	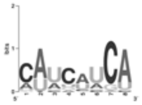	(WOLF *et al.* 2010)
Msl5	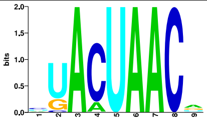	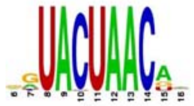	(GARREY *et al.* 2006)
Nab2	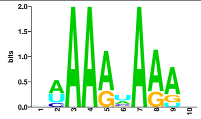	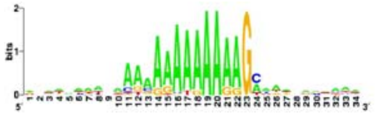	(KIM GUISBERT *et al.* 2005)
Nrd1	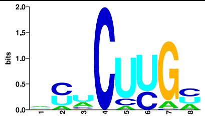	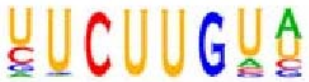	(HOGAN *et al.* 2008)
Pin4	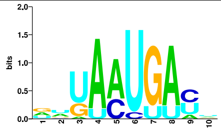	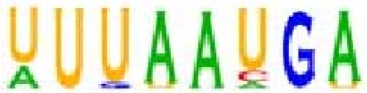	(HOGAN *et al.* 2008)
Pub1	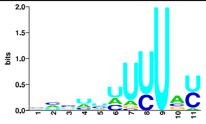	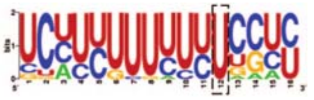	(DUTTAGUPTA *et al.* 2005)
Puf2	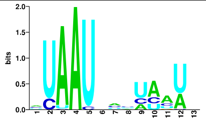	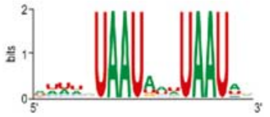	(YOSEFZON *et al.* 2011)
Puf3	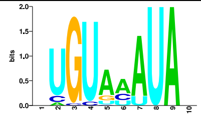	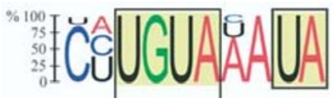	(GERBER *et al.* 2004)
Puf4	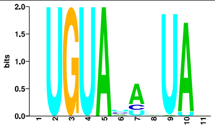	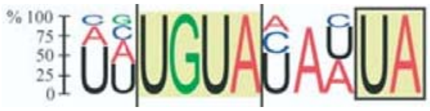	(GERBER *et al.* 2004)
Puf5	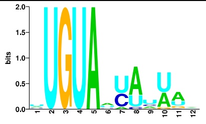	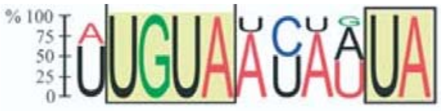	(GERBER *et al.* 2004)
Scp160	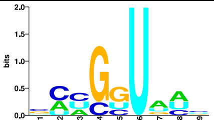	-	This study

Sik1	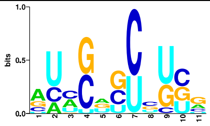	-	This study
Tdh3	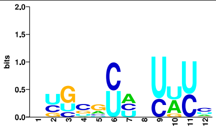	-	This study
YLL032C	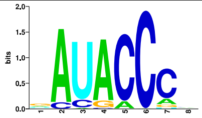	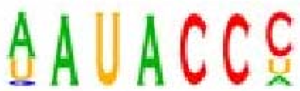	(HOGAN *et al.* 2008)

REFERENCES

Brachmann, C. B., A. Davies, G. J. Cost, E. Caputo, J. Li *et al.*, 1998 Designer deletion strains derived from Saccharomyces cerevisiae S288C: a useful set of strains and plasmids for PCR-mediated gene disruption and other applications. Yeast 14**:** 115-132.

Brem, R. B., G. Yvert, R. Clinton and L. Kruglyak, 2002 Genetic dissection of transcriptional regulation in budding yeast. Science 296**:** 752-755.

Duttagupta, R., B. Tian, C. J. Wilusz, D. T. Khounh, P. Soteropoulos *et al.*, 2005 Global analysis of Pub1p targets reveals a coordinate control of gene expression through modulation of binding and stability. Molecular and cellular biology 25**:** 5499-5513.

Garrey, S. M., R. Voelker and J. A. Berglund, 2006 An extended RNA binding site for the yeast branch point-binding protein and the role of its zinc knuckle domains in RNA binding. The Journal of biological chemistry 281**:** 27443-27453.

Gerber, A. P., D. Herschlag and P. O. Brown, 2004 Extensive association of functionally and cytotopically related mRNAs with Puf family RNA-binding proteins in yeast. PLoS biology 2**:** E79.

Hogan, D. J., D. P. Riordan, A. P. Gerber, D. Herschlag and P. O. Brown, 2008 Diverse RNA-binding proteins interact with functionally related sets of RNAs, suggesting an extensive regulatory system. PLoS biology 6**:** e255.

Kim Guisbert, K., K. Duncan, H. Li and C. Guthrie, 2005 Functional specificity of shuttling hnRNPs revealed by genome-wide analysis of their RNA binding profiles. RNA 11**:** 383-393.

Riordan, D. P., D. Herschlag and P. O. Brown, 2011 Identification of RNA recognition elements in the Saccharomyces cerevisiae transcriptome. Nucleic acids research 39**:** 1501-1509.

Smith, E. N., and L. Kruglyak, 2008 Gene-environment interaction in yeast gene expression. PLoS biology 6**:** e83.

Wolf, J. J., R. D. Dowell, S. Mahony, M. Rabani, D. K. Gifford *et al.*, 2010 Feed-forward regulation of a cell fate determinant by an RNA-binding protein generates asymmetry in yeast. Genetics 185**:** 513-522.

Yosefzon, Y., Y. Y. Koh, J. J. Chritton, A. Lande, L. Leibovich *et al.*, 2011 Divergent RNA binding specificity of yeast Puf2p. RNA 17**:** 1479-1488.
