# Supplementary material for: Harnessing Natural Sequence Variation to Dissect Posttranscriptional Regulatory Networks in Yeast
Source: G3 (Bethesda). 2014 Jun 17;4(8):1539–53. doi: 10.1534/g3.114.012039 (PMC4132183; doi:10.1534/g3.114.012039)
Supplement: Supporting Information [file supp_g3.114.012039_FigureS1.pdf]

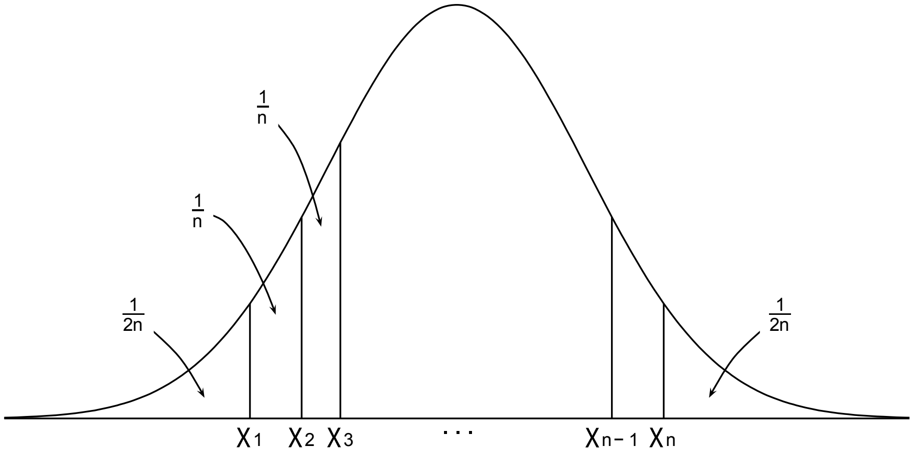

Figure S1 Schematic representation of the rank-quantile transformation step. The transformation is applied to each column (size n) of the binding data. We assigned i^{th}-quantile value (χ_i) to the ith element based on the rank of data point x_i.
