# Supplementary material for: Harnessing Natural Sequence Variation to Dissect Posttranscriptional Regulatory Networks in Yeast
Source: G3 (Bethesda). 2014 Jun 17;4(8):1539–53. doi: 10.1534/g3.114.012039 (PMC4132183; doi:10.1534/g3.114.012039)
Supplement: Supporting Information [file supp_g3.114.012039_FigureS4.pdf]

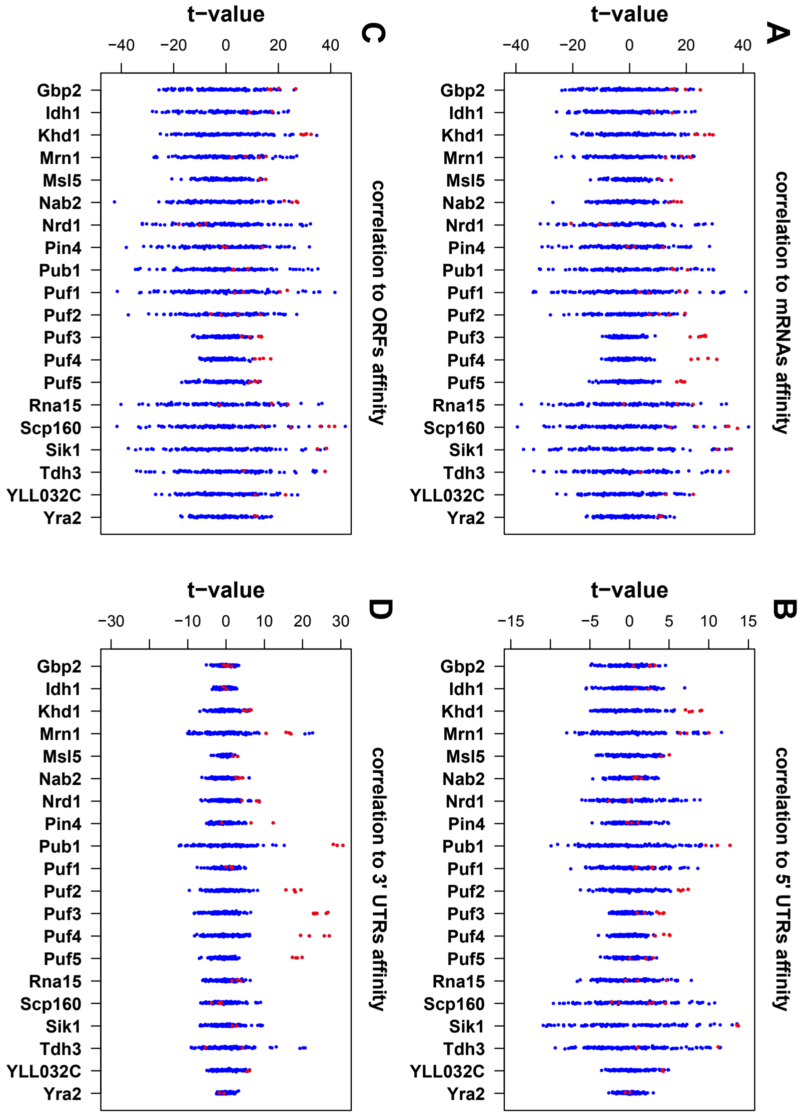


**Figure S4** Specificity test of all significant PSAMs derived for the RBPs. The scatter plots display the correlation of the RBP binding data to the affinity scores of the 20 PSAMs calculated on (A) complete mRNA sequence, (B) 5' UTRs, (C) ORFs, and (D) 3' UTRs.
