# Supplementary material for: Harnessing Natural Sequence Variation to Dissect Posttranscriptional Regulatory Networks in Yeast
Source: G3 (Bethesda). 2014 Jun 17;4(8):1539–53. doi: 10.1534/g3.114.012039 (PMC4132183; doi:10.1534/g3.114.012039)
Supplement: Supporting Information [file supp_g3.114.012039_FigureS5.pdf]

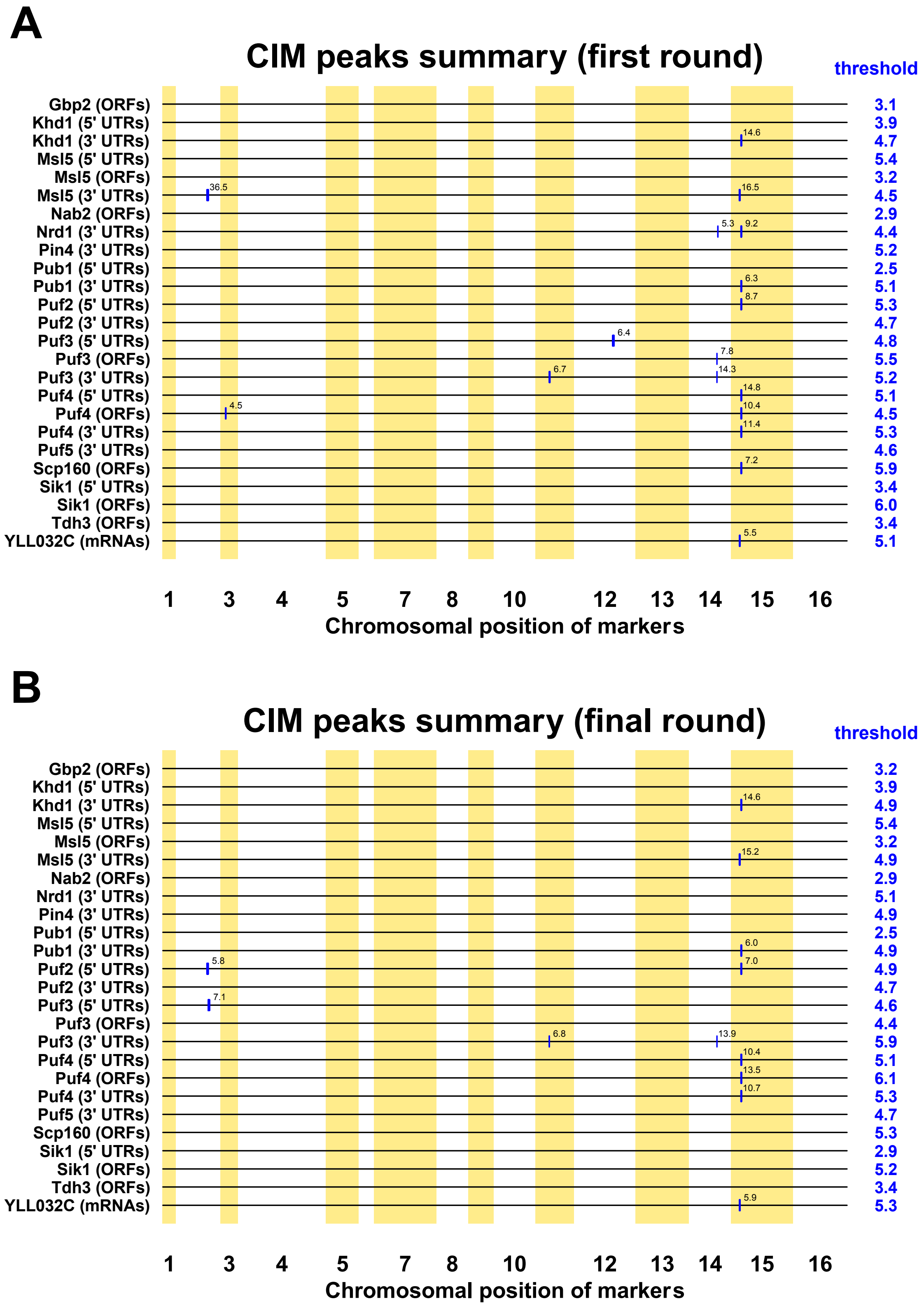

Figure S5 aQTL results for all of the 25 accepted RBP/feature combinations. (A) shows the significant peaks obtained by composite internal mapping (CIM) method when all genes were included (first round), and (B) shows the results after eliminating neighboring genes for each peak, genes with significant eQTL at these peaks and genes encoding the RBPs (last round). After 3 round of gene elimination, no new peak appeared.
