# Supplementary material for: Harnessing Natural Sequence Variation to Dissect Posttranscriptional Regulatory Networks in Yeast
Source: G3 (Bethesda). 2014 Jun 17;4(8):1539–53. doi: 10.1534/g3.114.012039 (PMC4132183; doi:10.1534/g3.114.012039)
Supplement: Supporting Information [file supp_g3.114.012039_FigureS6.pdf]

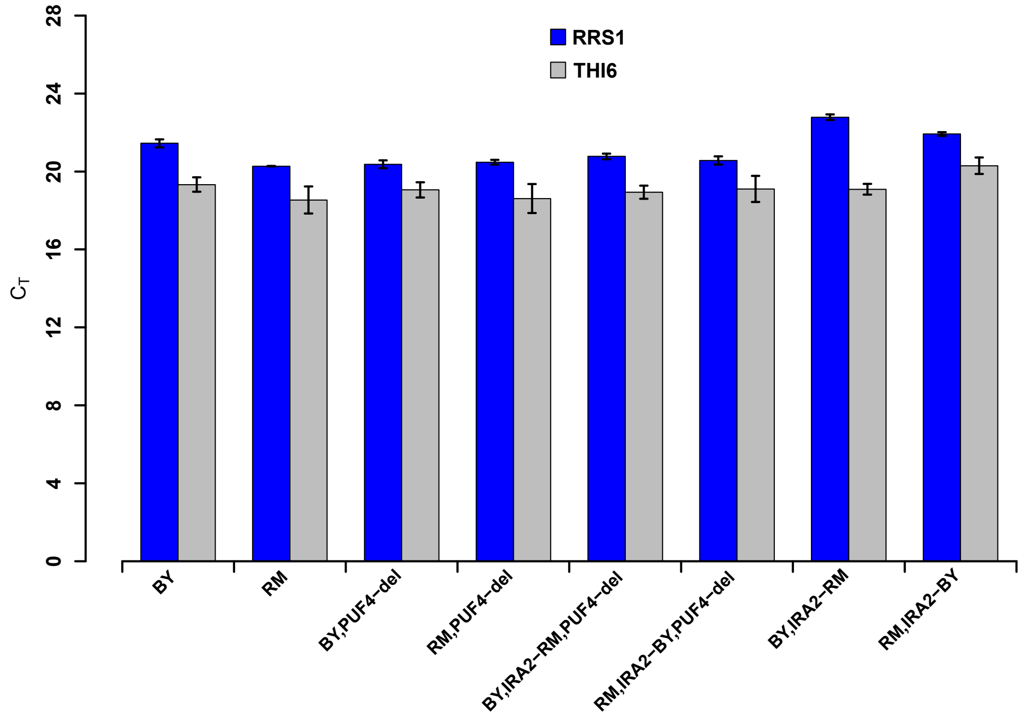

Figure S6 Barplot showing the C_T values for the qRT-PCR measurements of *RRS1* (test) and *THI6* (control) expression level. The mean and standard deviation are calculated based on the 3 technical replicates for each strain.
