# Supplementary material for: Harnessing Natural Sequence Variation to Dissect Posttranscriptional Regulatory Networks in Yeast
Source: G3 (Bethesda). 2014 Jun 17;4(8):1539–53. doi: 10.1534/g3.114.012039 (PMC4132183; doi:10.1534/g3.114.012039)
Supplement: Supporting Information [file supp_g3.114.012039_TableS1.pdf]

Table S1 PSAMs statistics on full data from optimization step (significant PSAMs)

RBP	mRNA region[a]	Pearson t-values on IP experiments	Spearman p-values on IP experiments	Accept[b]
Gbp2p	ORFs	25.3, 19.5, 15.8, 14.6	$1.2e^{-133}$, $3.1e^{-105}$, $1.9e^{-31}$, $4.0e^{-27}$	Yes
Idh1p	ORFs	9.1, 15.8	$2.7e^{-27}$, $1.3e^{-86}$	No
Khd1p	ORFs	27.2, 25.7, 28.5, 23.1, 22.2	$3.0e^{-89}$, $2.0e^{-74}$, $2.0e^{-109}$, $3.6e^{-68,}$ $1.0e^{-50}$	Yes
Mrn1p	mRNAs	21.8, 21.0, 18.5, 12.9	$1.3e^{-83}$, $3.0e^{-68}$, $1.8e^{-71}$, $8.4e^{-19}$	No
Msl5p	ORFs	9.3, 13.8	0.32, $1.8e^{-11}$	Yes
Nab2p	ORFs	23.8, 22.4, 21.3, 18.8	$1.7e^{-142}$, $1.5e^{-114}$, $3.3e^{-122}$, $3.4e^{-114}$	Yes
Nrd1p	3′ UTRs	4.2, 7.7, 8.4	$3.0e^{-8}$, $1.6e^{-13}$, $3.1e^{-16}$	Yes
Pin4p	3′ UTRs	6.3, -1.0, 11.5	$1.5e^{-7}$, $1.9e^{-4}$, $4.0e^{-15}$	Yes
Pub1p	3′ UTRs	28.3, 27.2, 30.0	$8.7e^{-143}$, $1.6e^{-145}$, $2.8e^{-152}$	Yes
Puf1p	ORFs	17.9, 3.3, 6.9, 20.3	$2.3e^{-65}$, $7.0e^{-25}$, $2.1e^{-19}$, $1.8e^{-89}$	No
Puf2p	3′ UTRs	15.6, 18.2, 17.6, 16.5	$9.0e^{-12}$, $2.4e^{-27}$, $6.8e^{-15}$, $1.3e^{-21}$	Yes
Puf3p	3′ UTRs	22.1, 22.8, 21.7, 25.0, 23.2	$1.1e^{-24}$, $1.6e^{-17}$, $2.2e^{-19}$, $8.0e^{-26}$, $1.6e^{-39}$	Yes
Puf4p	mRNAs	21.9, 30.9, 27.8, 24.3	$2.3e^{-73}$, $1.7e^{-123}$, $2.1e^{-73}$, $1.7e^{-43}$	Yes
Puf5p	mRNAs	19.6, 16.8, 18.2, 18.9	$4.6e^{-67}$, $1.0e^{-37}$, $3.5e^{-30}$, $3.5e^{-38}$	Yes
Rna15p	ORFs	16.6, 22.1, -2.4	$3.1e^{-89}$, $1.5e^{-136}$, $1.4e^{-5}$	No
Scp160p	ORFs	15.2, 24.7, 35.9, 38.7, 35.1	$1.9e^{-72}$, $4.4e^{-166}$, $4.4e^{-290}$, 0, 0	Yes
Sik1p	5′ UTRs	13.7, 13.9	$1.4e^{-49}$, $9.1e^{-62}$	Yes
Tdh3p	ORFs	34.0, 5.6	$9.0e^{-272}$, $7.9e\text{-}^{16}$	Yes
YLL032C	mRNAs	22.7, 13.0	$3.7e^{-112}$, $6.1e^{-19}$	Yes
Yra2p	mRNAs	11.4, 10.3	$2.4e^{-14}$, $1.9e^{-11}$	No

[a] represents the region (complete mRNA, 5′ UTR, ORF, or 3′ UTR) the PSAM was trained on

[b] acceptance based on the specificity test
