# Supplementary material for: Harnessing Natural Sequence Variation to Dissect Posttranscriptional Regulatory Networks in Yeast
Source: G3 (Bethesda). 2014 Jun 17;4(8):1539–53. doi: 10.1534/g3.114.012039 (PMC4132183; doi:10.1534/g3.114.012039)
Supplement: Supporting Information [file supp_g3.114.012039_TableS4.pdf]

Table S4 List of genotype of strains used in RT-PCR.

Strain	Parent	Background	Genotype	Reference
BY4716	BY	S288c	*MATα lys2Δ0*	(BRACHMANN *et al.* 1998)
RM11-1a	RM11-1a	RM11-1a	*MATa leu2Δ0 ura3Δ0 ho::KanMX*	(BREM *et al.* 2002)
	BY4741	S288c	*MATa his3Δ1 leu2Δ0 met15Δ0 ura3Δ PUF4::KanMX*	Research Genetics Deletion Collection
IMY204	BY4716	S288c	*MATα lys2Δ0 PUF4::KanMX*	This Study
YLK807/HCY475	BY4724	S288c	*MATa lys2Δ0 ura3Δ0 IRA2RM*	(SMITH and KRUGLYAK 2008)
IMY207	YLK807/ HCY475	S288c	*MATa lys2Δ0 ura3Δ0 IRA2RM PUF4::KanMX*	This study
IMY224	RM11-1a	RM11-1a	*MATa leu2Δ0 ura3Δ0 ho::NatMX*	This study
IMY229	IMY224	RM11-1a	*MATa leu2Δ0 ura3Δ0 ho::NatMX PUF4::KanMX*	This study
YLK810/IMY300	RM11-1a	RM11-1a	*MATa leu2Δ0 ura3Δ0 ho::KanMX IRA2BY*	(SMITH and KRUGLYAK 2008)
IMY236	IMY220	RM11-1a	*MATa leu2Δ0 ura3Δ0 ho::NatMX IRA2BY PUF4::KanMX*	This study
