# Supplementary material for: Harnessing Natural Sequence Variation to Dissect Posttranscriptional Regulatory Networks in Yeast
Source: G3 (Bethesda). 2014 Jun 17;4(8):1539–53. doi: 10.1534/g3.114.012039 (PMC4132183; doi:10.1534/g3.114.012039)
Supplement: Supporting Information [file supp_g3.114.012039_TableS5.pdf]

Table S5 Motifs obtained by MatrixREDUCE and reported in the literature for 15 RBPs.

RBP	MatrixREDUCE motif	Reported motif in literature	References
Gbp2	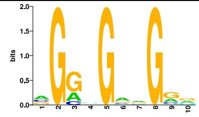	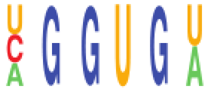	(RIORDAN *et al.* 2011)
Khd1	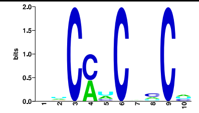	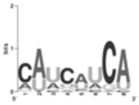	(WOLF *et al.* 2010)
Msl5	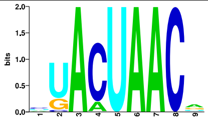	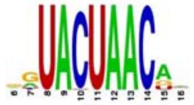	(GARREY *et al.* 2006)
Nab2	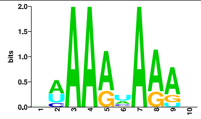	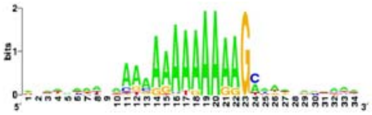	(KIM GUISBERT *et al.* 2005)
Nrd1	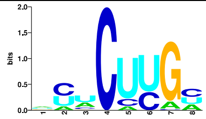	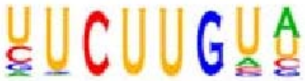	(HOGAN *et al.* 2008)
Pin4	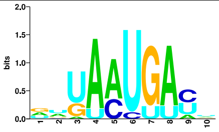	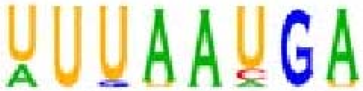	(HOGAN *et al.* 2008)
Pub1	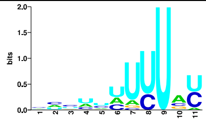	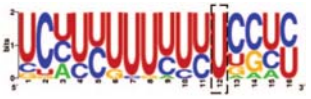	(DUTTAGUPTA *et al.* 2005)
Puf2	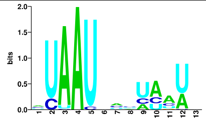	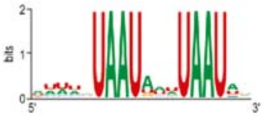	(YOSEFZON *et al.* 2011)
Puf3	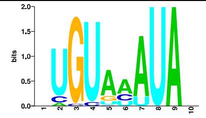	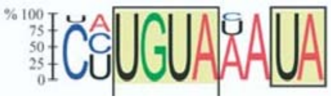	(GERBER *et al.* 2004)
Puf4	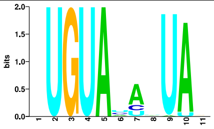	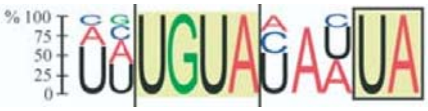	(GERBER *et al.* 2004)
Puf5	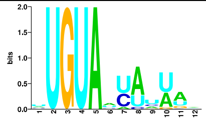	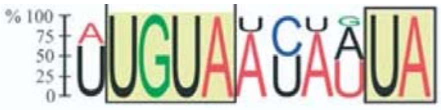	(GERBER *et al.* 2004)
Scp160	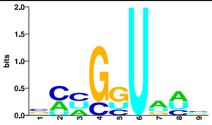	-	This study

Sik1	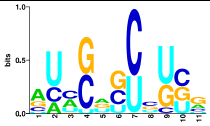	-	This study
Tdh3	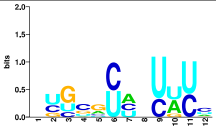	-	This study
YLL032C	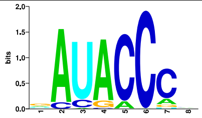	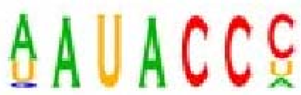	(HOGAN *et al.* 2008)
